# Anticipating future learning affects current control decisions: A comparison between passive and active adaptive management in an epidemiological setting

**DOI:** 10.1016/j.jtbi.2020.110380

**Published:** 2020-12-07

**Authors:** Benjamin D. Atkins, Chris P. Jewell, Michael C. Runge, Matthew J. Ferrari, Katriona Shea, William J.M. Probert, Michael J. Tildesley

**Affiliations:** a Mathematics for Real-World Systems Centre for Doctoral Training, Mathematics Institute, University of Warwick, Coventry CV4 7AL, United Kingdom; b Lancaster Medical School, Lancaster University, Lancaster LA1 4YG, United Kingdom; c U.S. Geological Survey, Patuxent Wildlife Research Center, Laurel, MD 20708, USA; d The Center for Infectious Disease Dynamics and Department of Biology, The Pennsylvania State University, University Park, PA 16802, USA; e Big Data Institute, Nuffield Department of Medicine, University of Oxford, Oxford OX37LF, United Kingdom; f Zeeman Institute: Systems Biology and Infectious Disease Epidemiology Research (SBIDER), Mathematics Institute and School of Life Sciences, University of Warwick, Coventry CV4 7AL, United Kingdom

**Keywords:** Infectious disease outbreaks, Optimal control, Uncertainty resolution, Real-time decision-making

## Abstract

Infectious disease epidemics present a difficult task for policymakers, requiring the implementation of control strategies under significant time constraints and uncertainty. Mathematical models can be used to predict the outcome of control interventions, providing useful information to policymakers in the event of such an epidemic. However, these models suffer in the early stages of an outbreak from a lack of accurate, relevant information regarding the dynamics and spread of the disease and the efficacy of control. As such, recommendations provided by these models are often incorporated in an *ad hoc* fashion, as and when more reliable information becomes available. In this work, we show that such trial-and-error-type approaches to management, which do not formally take into account the resolution of uncertainty and how control actions affect this, can lead to sub-optimal management outcomes. We compare three approaches to managing a theoretical epidemic: a non-adaptive management (AM) approach that does not use real-time outbreak information to adapt control, a passive AM approach that incorporates real-time information if and when it becomes available, and an active AM approach that explicitly incorporates the future resolution of uncertainty through gathering real-time information into its initial recommendations. The structured framework of active AM encourages the specification of quantifiable objectives, models of system behaviour and possible control and monitoring actions, followed by an iterative learning and control phase that is able to employ complex control optimisations and resolve system uncertainty. The result is a management framework that is able to provide dynamic, long-term projections to help policymakers meet the objectives of management. We investigate in detail the effect of different methods of incorporating up-to-date outbreak information. We find that, even in a highly simplified system, the method of incorporating new data can lead to different results that may influence initial policy decisions, with an active AM approach to management providing better information that can lead to more desirable outcomes from an epidemic.

## Highlights

• Adaptive epidemic control.
• Using real-time outbreak information to improve epidemic control.
• Active Adaptive Management in an epidemiological setting.
• Analysing the interaction between control and monitoring during an epidemic.

## 1 Introduction

The management of infectious disease epidemics is a task beset by difficulties. It requires satisfying the complex, and often conflicting, objectives of stakeholders, without complete knowledge of how the disease will spread or the effect of control. Mathematical models have become a useful tool to aid in the decision-making process, allowing the comparison of different strategies through simulation (Keeling et al., 2001, Ferguson et al., 2001, Keeling et al., 2003, Tildesley et al., 2006, Shea et al., 2014, Bradbury et al., 2017, Probert et al., 2018, Li et al., 2017). However, incomplete knowledge of the system can be a significant barrier to providing relevant policy recommendations (Tildesley et al., 2006, Shea et al., 2014, Bradbury et al., 2017, Probert et al., 2018, Li et al., 2017, O’Neill et al., 1999, Lekone et al., 2006, Cauchemez and Ferguson, 2008, Jewell et al., 2009,, Jewell et al., 2009, Elderd et al., 2006). In the context of a novel outbreak, control strategies must be decided upon and implemented quickly, leaving little time to gather accurate information about the current outbreak, such as the length of time an individual remains infectious or the efficacy of a vaccine. As a result, the retrospective analyses of historic outbreaks are often used to estimate such parameters. In such instances, real-time information then may be ignored or used in an *ad hoc* fashion as the outbreak progresses.

The adaptive management (AM) framework, more specifically ‘active’ AM, has been proposed as a way to tackle some of the problems posed by system uncertainty in epidemic management (Merl et al., 2009, Shea et al., 2014, Bradbury et al., 2017, Li et al., 2017). Active AM is an iterative, structured approach to decision-making that provides information that can lead to dynamic, state-dependent decision recommendations, encouraging the incorporation of real-time outbreak information to resolve uncertainty in system parameters where necessary (Probert et al., 2011, Westgate et al., 2013, Williams et al., 2011, Allen et al., 2011). It is well-established in ecological fields, such as conservation and resource management (Holling et al., 1978, Hilborn et al., 1988, Walters, 1986), and has recently gained attention in the literature surrounding epidemiological interventions (Bradbury et al., 2017, Shea et al., 2014, Li et al., 2017). However, widespread, interdisciplinary use has been hindered by a lack of consensus on the definition of AM and a lack of understanding as to how active AM differs from current methods of management, such as *ad hoc*, trial-and-error type approaches (Allen et al., 2011, Westgate et al., 2013, Probert et al., 2011).

A major distinction between active AM and other approaches is that it explicitly accounts for the effect that resolving uncertainty will have on our ability to make optimal decisions. Under active AM, we are able to observe that, with more information, the predicted efficacy of competing policies can change significantly and a different policy may become optimal. There are therefore circumstances where it may be beneficial to choose an intervention policy that allows for a rapid resolution of system uncertainty, as opposed to a policy that may appear to provide better control under the current level of information, but hinders the gathering of real-time information.

In this work, we investigate how different methods of including real-time information can affect policy selection during an epidemic and, in turn, how this affects our ability to satisfy the objectives of management. We compare three approaches to managing a theoretical epidemic: non-AM, passive AM and active AM. The epidemic is represented by a deterministic, non-spatial Susceptible-Exposed-Infected-Recovered (SEIR) compartmental model, with control limited to vaccination of the susceptible population at a fixed daily vaccination rate, restricted by a finite vaccine pool. There is no uncertainty regarding the spread of the disease in the absence of control, however a single source of uncertainty is introduced through an unknown vaccine efficacy, defined as the probability that an administered vaccine will result in immunity. Information regarding the vaccine efficacy can be collected throughout the outbreak by monitoring a proportion of administered vaccines for success: a successful vaccination results in complete, indefinite immunity that takes effect, and can be tested, immediately. Conversely, we assume that unsuccessful vaccinations result in no immunity.

The non-AM approach represents a static control policy, in which real-time information is not used to improve control. Under this approach, there is a single decision opportunity at the start of the outbreak (day 0), for which we must decide whether to implement a vaccination campaign or not. Hence, this approach results in either a static vaccination campaign that is implemented immediately and continued until the vaccine pool is depleted, or no vaccination throughout. This approach provides a baseline for the performance of control, or no control. The passive and active AM approaches allow for adaptation of control on a single, predetermined day during the outbreak ($t^{*}$). For these approaches, there are two decision opportunities: the initial decision on day 0 and a final decision on day $t^{*}$. The initial decision is whether or not to implement a vaccination campaign immediately and continue it until at least day $t^{*}$. The final decision is whether or not to vaccinate from day $t^{*}$ until the vaccine pool is depleted (i.e. continue an ongoing campaign, stop an ongoing campaign, start a campaign or continue with no campaign). On day 0, for the initial decision, we have only the ‘prior information’ regarding vaccine efficacy. On day $t^{*}$, the results of monitored vaccinations, if any vaccinations have been administered, are used to provide updated information regarding vaccine efficacy. Hence, an initial decision to vaccinate allows for the resolution of uncertainty in vaccine efficacy, whilst an initial decision not to vaccinate does not. Passive AM does not incorporate the effect of reducing uncertainty in the vaccine efficacy into the initial decision, hence, whilst this information might be used for the final decision, we do not plan to use it. As such, passive AM represents a reactive approach to incorporating real-time information. Active AM explicitly incorporates the resolution of uncertainty when assessing the initial decision. Therefore, if choosing to vaccinate, thereby allowing uncertainty in vaccine efficacy to be reduced, leads to significantly improved future management, active AM will incorporate this information and the results will support a decision to vaccinate immediately.

For the basis of this analysis, we focus on two scenarios, contrasted primarily by different management objectives. Scenario 1: we allocate a ‘cost’ to the epidemic, defined by a linear combination of the number of infections, vaccines administered and a fixed cost associated with implementing a vaccination campaign. The objective of management is to minimise the expected value of this cost. This scenario could be likened to a non-fatal, human disease context, or livestock disease context, where the cost of implementing a vaccination campaign must be weighed against the expected benefits resulting from the campaign. In this scenario, we parametrise the epidemic model using flu-like transmission, incubation and recovery rates, with a basic reproductive number ($R_{0}$) of 1.6. The relative weights of infections and vaccinations in the calculation of cost are fixed for the main result, however the effect these have on the result is explored in detail in the subsequent sensitivity sections.

Scenario 2: the objective of management is to minimise the expected duration of the outbreak, regardless of the number of infections caused or vaccines used. This scenario could be likened to a livestock disease context in which there are significant daily costs to the economy caused by an ongoing outbreak, such as loss of exports or tourism. In such a context, regaining a ‘disease-free’ status as quickly as possible may be the primary concern. In this scenario we parametrise the epidemic model with Foot-and-Mouth-like transmission, incubation and recovery rates, with an $R_{0}$ of 2. The control restrictions (daily vaccination rate, vaccine pool and $t^{*}$) used in each scenario are summarised in Table A.2, alongside other fixed parameters. For both scenarios, the effects of changing the epidemiological parameters used and the restrictions on control are explored in detail in the sensitivity sections. The treatment of such parameters in a real-world scenario, which are likely to be uncertain rather than fixed, is addressed in the discussion.

For both scenarios, we initially assume a large amount of uncertainty in the vaccine efficacy at the start of the outbreak, or equivalently, a very low amount of prior information, defined by a Beta distribution centred around 50% efficacy with a high variance (Methods; Prior and real-time information). The effect of having more prior information to inform our decisions is also explored in detail.

We investigate the performances of the three approaches for the two specific scenarios, showing that the method of incorporating real-time information can have a significant effect. We also show how their performance changes under different conditions, varying the amount of prior and real-time information available from the outbreak, restrictions on control and epidemiological parameters. Our focus on how passive approaches can lead to different results affecting control recommendations compared to active approaches, specifically in the context of infectious disease epidemics, extends similar explorations in both the ecological and epidemiological literature (Shea et al., 2014, Moore et al., 2017). Overall, we see that even highly simplified systems can be difficult to control in the presence of uncertainty and the method of incorporating real-time information into management decisions can have a significant effect on policy selection. We find that active AM is best able to provide information to meet management objectives, whilst also providing more usable information to decision-makers with regards to the collection of real-time information and the timing and delivery of control.

## 2 Materials and methods

### 2.1 Adaptive management

**Fig. 1:**
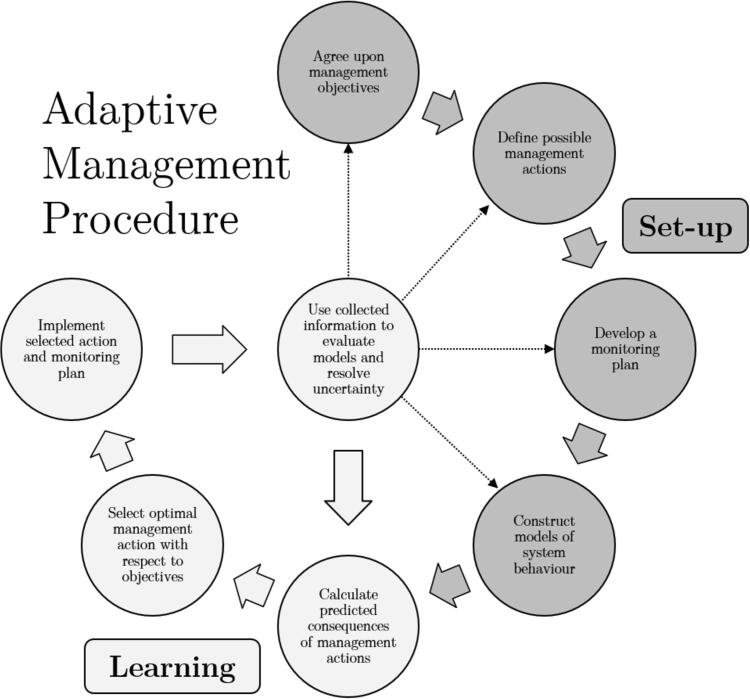
**Adaptive management procedure.** Adaptive management (AM) addresses the difficulties of epidemic control through a structured, iterative framework. The set-up phase (dark grey circles) provides a quantitative representation of management objectives, possible actions, planned monitoring and system behaviour, decided upon *a priori* with input from stakeholders. In the learning and implementation phase, the set-up components are used to forecast the possible effects of control. This allows a dynamic policy recommendation, outlining the actions that will best satisfy the management objectives whilst predicting the possible effect of updated outbreak information on the efficacy of control. As the epidemic progresses, predictions from competing models of system behaviour are compared to incoming information, reducing uncertainty in the effect of control. The recommendations are adapted as necessary and the process repeats. Figure adapted from (Allen et al., 2011).

We provide a brief overview of the AM framework, in the context of epidemic interventions, in Fig. 1. See also Table A.1 in Appendix A. Similar applications in the literature include (Shea et al., 2014, Bradbury et al., 2017, Li et al., 2017). For a more detailed review of the AM framework in general we refer the reader to (Allen et al., 2011, Westgate et al., 2013, Holling et al., 1978, Walters, 1986, Hilborn et al., 1988, Probert et al., 2011).

### 2.2 System representation

We describe the spread of a directly transmitted disease throughout a population via a non-spatial, homogeneously mixing, deterministic SEIR (Susceptible, Exposed, Infected, Recovered/Removed) model, with constant transmission rate (β), incubation rate (σ) and recovery/removal rate (γ; this can include both recovery and death from the disease). We ignore demography on the assumption that the dynamics of the epidemic are significantly faster than the natural birth–death process of the population. The transmission, incubation and recovery rates used in each scenario are provided in Table A.2. For both scenarios, the initial population is made up of 5000 susceptible and 1 infected individual. We assume that the epidemic is not detected until the number of infected individuals reaches 20. We denote *t* as the number of days since the epidemic was detected, with t=0 representing the day of detection and initial management decision.

Control is limited to vaccination of the susceptible population. We assume that vaccinations are perfectly targeted towards susceptible individuals, excluding the exposed class from vaccination, to help clarify the link between uncertainty in vaccine efficacy and the predicted outcome of control. Vaccinations can occur at a constant daily rate ($\nu_{r}$; number of vaccinations per day), restricted by a limit on the total number of vaccines available ($\nu_{\mathrm{pool}}$). The vaccine is assumed to result in immediate and indefinite immunity, with probability $\nu_{e}$. This probability, the ‘vaccine efficacy’, is unknown and provides the only source of uncertainty in the system.

A detailed, mathematical representation of the model can be found in Appendix A.2, along with a summary of parameters and notation used (Table A.2).

### 2.3 Objectives of management

The objective of management differs between the two scenarios, however can be summarised in general terms by defining a flexible cost function that incorporates multiple factors: the duration of the outbreak, the number of vaccines administered, a fixed cost associated with implementing a vaccination campaign and the number of infections caused by the epidemic. In both scenarios, the objective of management is to optimise the expected outcome of the outbreak, over the unknown vaccine efficacy. The cost function and calculation of the expected outcome are detailed mathematically in Appendix A.3.

### 2.4 Prior and real-time information

Prior and real-time information focuses on resolving the uncertainty in the system introduced by an unknown vaccine efficacy ($\nu_{e}$). We define this information in a quantitative manner using a *Beta* distribution (see Appendix A.4 for details). This allows us to define a mode of the distribution, representing an estimate of vaccine efficacy, and also a measure of how much weight we are giving to this estimate. In general, throughout we use a prior distribution centred around 50% efficacy with a large variance, representing a situation where we do not have a strong idea of what the efficacy is, but are aware that it is less likely to be completely effective (100% efficacy) or completely ineffective (0% efficacy).

Real-time information is collected throughout the outbreak by monitoring a proportion (ρ) of administered vaccinations for success. We assume that the success or failure of a vaccine can be tested immediately after it is administered and this test will always give the true result. Whilst this is an unrealistic assumption, it allows us to clearly identify the relationship between monitored vaccinations and the resolution of uncertainty in vaccine efficacy. This real-time information is combined with the prior information using a Bayesian approach, to give a posterior distribution around the vaccine efficacy, also defined by a *Beta* distribution.

A detailed mathematical description of this process is provided in Appendix A.4.

### 2.5 Decision making approaches

In this work, we compare three approaches to decision making during the outbreak: non-AM, passive AM and active AM. These approaches are contrasted by how they incorporate real-time outbreak information, in this case the results of monitored vaccinations. Under any of the three approaches, at each decision point we must choose to vaccinate until the next decision point or not. If there are no future decision points, this equates to vaccinating until the vaccine pool is depleted, or forgoing vaccination for the remainder of the epidemic. We allow a maximum of two decision points (one for the non-AM approach): an initial decision is made when the outbreak is detected (t=0) and a final decision is made on a predetermined day during the outbreak ($t=t^{*}$).

Under the non-AM approach we allow only the initial decision. Under the adaptive approaches, a proportion of the vaccines administered between the initial and final decision points, if any, are monitored for success and this information is used to inform the final decision. The adaptive approaches differ in how they make the initial decision. Passive AM ignores the effect that updated vaccine efficacy information may have on future decisions, hence assumes that future decisions will be made using only our prior level of knowledge. However, active AM explicitly accounts for real-time information regarding vaccine efficacy and anticipates how different outcomes from monitored vaccinations may change our decisions in the future. The method of decision making at both decision points, under each management approach, is formalised mathematically in Appendix A.5.

## 3 Results and discussion

### 3.1 Scenario 1

In the first scenario, we test our ability to minimise the ‘cost’ of a theoretical epidemic. Cost is defined as a linear combination of the number of infections caused by the outbreak, number of vaccines administered and a fixed cost associated with implementing a vaccination campaign (if one is implemented). The weight of each contributing factor is defined relative to that of a single infection, hence $\omega_{2}=1$ (see Appendix A.3). For the majority of the analyses undertaken under this scenario we set the relative costs of vaccination to 0.6 per vaccine ($\omega_{3}=0.6$) plus a fixed cost of 350 ($\omega_{4}=350$) and assume that the epidemiological parameters are representative of a flu-like disease such that the transmission rate β=0.23, the incubation rate σ=0.5 and the removal/recovery rate γ=0.14, with $R_{0}=1.6$. Vaccination is limited to 100 individuals per day, with a total pool of 2500 vaccines. These parameters are summarised in Table A.2. Sensitivity to all these parameters is explored in detail in later sections.

**Fig. 2:**
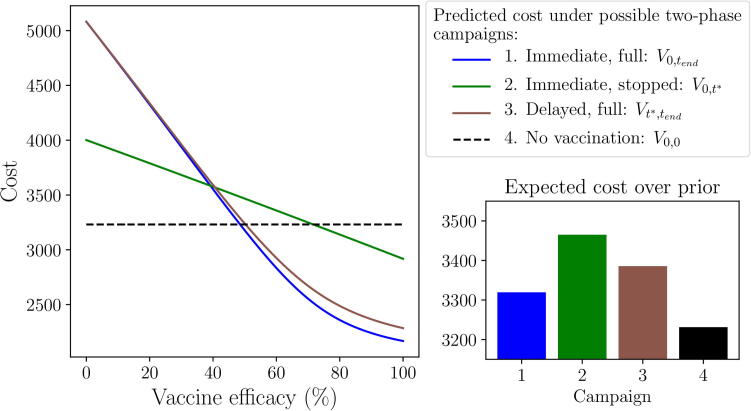
**Scenario 1: Predicted outbreak cost resulting from possible two-phase campaigns.** For the passive and active AM methods, the two decision points ($t=\{0,t^{*}\}$) result in four possible two-phase campaigns that may be implemented by the end of the outbreak: 1) vaccination is started immediately and continued until the vaccine pool is depleted, 2) vaccination is started immediately but stopped on day $t^{*}$, 3) vaccination is delayed until $t^{*}$, then continued until the vaccine pool is depleted, or 4) no vaccination is employed during the outbreak. The non-AM approach has only one decision point (t=0), hence can only result in either campaign (1) or (4). Epidemiological and vaccination parameters are set to those in Table A.2: Scenario 1. Expected cost is calculated over a *Beta*(1.1,1.1) prior distribution around vaccine efficacy (see Appendix A).

With a maximum of two decision points ($t=\{0,t^{*}\}$), there are a maximum of four possible campaigns that may be implemented by the end of the outbreak (Fig. 2): 1) $V_{0,t_{\mathrm{end}}}$, vaccination is implemented immediately and continued until the vaccine pool is depleted, 2) $V_{0,t^{*}}$, vaccination is implemented immediately and stopped on day $t^{*}$, 3) $V_{t^{*},t_{\mathrm{end}}}$, vaccination is delayed until day $t^{*}$, then performed until the vaccine pool is depleted, and 4) $V_{0,0}$, no vaccines are administered during the outbreak. Under active and passive AM, all four of these campaigns are taken into consideration, whilst under the non-AM approach only campaigns (1) and (4) are considered.

**Fig. 3:**
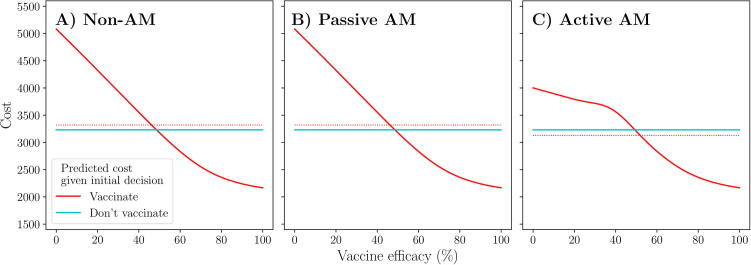
**Scenario 1: Predicted outbreak cost resulting from an initial decision to vaccinate or not.** Predicted outbreak cost from implementing vaccination immediately (red) or not (blue), as viewed under non-AM (A), passive AM (B) and active AM (C) methods. Dotted lines represent the expected cost, calculated over a *Beta*(1.1,1.1) prior distribution around vaccine efficacy (see Appendix A.4). Epidemiological and vaccination parameters are set to those in Table A.2: Scenario 1.

Under the non-AM approach to management, the initial decision to vaccinate or not cannot be adapted. Hence, an initial decision to vaccinate is equivalent to committing to a full vaccination campaign ($V_{0,t_{\mathrm{end}}}$; Fig. 3 red line) and an initial decision not to vaccinate is equivalent to foregoing vaccination for the duration of the outbreak ($V_{0,0}$; Fig. 3 blue line). Thus, under this approach, the optimal policy is to not vaccinate initially, and throughout, since it provides a lower expected cost over the prior distribution around vaccine efficacy than a full campaign. There is no opportunity to adapt this, hence we would forego vaccination for the duration of the outbreak under this approach.

Under passive AM, we recognise that an initial decision to vaccinate or not can be adapted on day $t^{*}$. Passive AM plans for this adaptation based on the prior information regarding vaccine efficacy. Hence, an initial decision to vaccinate is assumed to lead to a final decision to also vaccinate, since, over the prior distribution, stopping the campaign on day $t^{*}$ would lead to a higher expected cost than continuing it (Fig. 2). Thus, the expected cost of an initial decision to vaccinate is equivalent to the expected cost of an immediate, full campaign ($V_{0,t_{\mathrm{end}}}$) under passive AM (Fig. 3 red line). Similarly, an initial decision not to vaccinate is assumed to always result in no vaccination throughout the outbreak, since a delayed, full campaign ($V_{t^{*},t_{\mathrm{end}}}$) results in a higher expected cost than no vaccination throughout ($V_{0,0}$) over the prior distribution (Fig. 2). Hence, under passive AM, the expected cost of an initial decision not to vaccinate is equivalent to the expected cost of foregoing vaccination completely (Fig. 3 blue line). Therefore, the optimal policy for passive AM is an initial decision not to vaccinate, since the expected cost of not vaccinating throughout is less than the expected cost of an immediate, full campaign. Since we do not have any vaccinations to monitor, no new information is available on day $t^{*}$ and thus the final decision will also be to not vaccinate.

Under active AM, we again recognise that an initial decision to vaccinate or not can be adapted on day $t^{*}$, but also that this will depend on the results of monitored vaccinations. Hence, an initial decision to vaccinate is assumed to lead to continued vaccination if the success rate from monitored vaccinations is sufficiently high (larger than approximately 40%, based on the parameters we have chosen), otherwise vaccination will be stopped on day $t^{*}$. Thus, the expected cost of an initial decision to vaccinate is derived from a combination of campaigns 1 and 2 (Fig. 3 red line): if the true vaccine efficacy is low, we are likely to get a low success rate from monitored vaccinations and stop the campaign, whereas if true efficacy is high the opposite will occur. Close to the value of vaccine efficacy where the cost of stopping and continuing the campaign cross over (approximately 40%), there is still uncertainty as to which choice is optimal even with the results from monitored vaccinations, hence the expected cost is increased slightly by the possibility of making the wrong decision. In contrast, an initial decision not to vaccinate results in there being no vaccinations to monitor. Hence, as under passive AM, the expected cost of such an initial decision is equivalent to the expected cost of foregoing vaccination for the entire outbreak ($V_{0,0}$; Fig. 3 blue line). The optimal policy for active AM is to vaccinate initially, since the benefit from learning, and the ability to stop the campaign if vaccine efficacy is proving to be low, outweighs the perceived benefit of not vaccinating at all. In this scenario, if we were to implement this policy, it would result in 35 monitored vaccinations by day $t^{*}$. If at least 14 of these result in immunity, we would continue vaccination on day $t^{*}$, otherwise we would stop the campaign.

In summary, we observe that the three methods of incorporating the information from monitored vaccinations result in different management decisions. The optimal policy for both a non-AM and passive AM approach is to forego vaccination for the entirety of the outbreak, since, under the prior distribution, the expected benefit of a full vaccination campaign is not sufficient to offset the cost of the vaccines. However, under active AM, we recognise that an ineffective campaign can be stopped on day $t^{*}$, saving the cost of administering the remaining vaccines and thus lowering the overall expected cost of immediate vaccination. Hence, the optimal policy for active AM is to start vaccination immediately and continue until the vaccine pool is depleted if monitored vaccinations are successful (in this scenario, if at least 14 of the 35 monitored vaccinations are successful), otherwise cease vaccination on day $t^{*}$. In this scenario, by incorporating the possible future results of monitored vaccinations into our initial decision, following active AM would reduce the expected cost of the outbreak by over 100 units (approximately 3%) compared to following a passive or non-AM approach. Hence, only active AM truly satisfies our management objective of minimising expected outbreak cost.

### 3.2 Scenario 2

In the second scenario we focus on our ability to minimise the duration of a theoretical epidemic ($\omega_{1}=1,\omega_{2}=\omega_{3}=\omega_{4}=0$; see Appendix A.3). Such an objective that may be suitable for some livestock disease epidemics, for which eradicating the disease as quickly as possible is the primary concern, in order to minimise the impact on the economy through exports and tourism. We parameterise the epidemiological model using FMD-like parameters; transmission: β=0.2, incubation: σ=0.2 and removal/recovery: γ=0.1, with $R_{0}=2$. Vaccination is limited to 100 individuals per day, with a total pool of 4500 vaccines. These are summarised in Table A.2. Sensitivity to all these parameters is explored in detail in later sections.

**Fig. 4:**
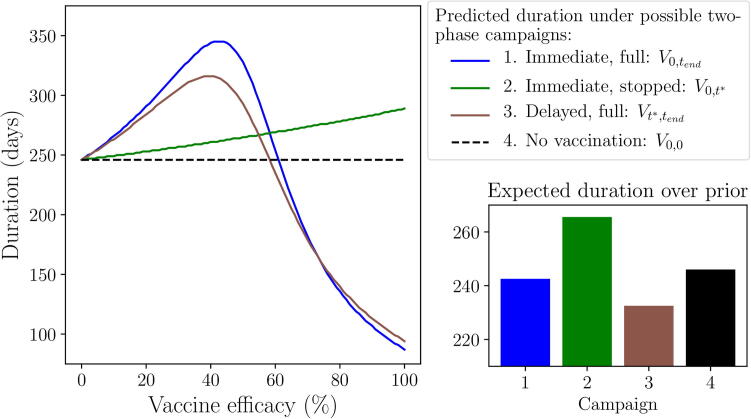
**Scenario 2: Predicted outbreak duration resulting from possible two-phase campaigns.** For the passive and active AM methods, the two decision points ($t=\{0,t^{*}\}$) result in four possible two-phase campaigns that may be implemented by the end of the outbreak: 1) vaccination is started immediately and continued until the vaccine pool is depleted, 2) vaccination is started immediately but stopped on day $t^{*}$, 3) vaccination is delayed until $t^{*}$, then continued until the vaccine pool is depleted, or 4) no vaccination is employed during the outbreak. The non-AM approach has only one decision point (t=0), hence can only result in either campaign (1) or (4). Epidemiological and vaccination parameters are set to those in Table A.2: Scenario 2. Expected duration is calculated over a *Beta*(1.1,1.1) prior distribution around vaccine efficacy (see Appendix A.4).

As in scenario 1, there are a maximum of four possible campaigns that may be implemented by the end of the outbreak (Fig. 4). Compared to scenario 1, the behaviour of the objective over the range of vaccine efficacy in this scenario is less intuitive. Here, if vaccine efficacy is low or too few vaccines are administered, we may see an increase in outbreak duration compared to taking no action. This occurs if the vaccination campaign is not sufficient to reduce the effective $R_{0}$ of the epidemic below 1 before it is stopped, leading to a longer, albeit much smaller, outbreak. Another consequence of this is that a delayed campaign (3: $V_{t^{*},t_{\mathrm{end}}}$) can be more effective at shortening duration than an immediate campaign, since a delayed campaign allows the disease to spread unhindered for 7 days before vaccination is implemented, hence the outbreak burns through the population faster. In scenario 1, when the number of infections was important not duration, a delayed campaign was never considered more effective than an immediate one.

Under the non-AM approach, we only compare campaigns (1: $V_{0,t_{\mathrm{end}}}$) and (4: $V_{0,0}$): immediate, full vaccination or no vaccination respectively. The expected duration over the prior distribution around vaccine efficacy is lower for the former, hence the optimal policy for this approach is to vaccinate immediately and continue vaccination until the vaccine pool is depleted.

**Fig. 5:**
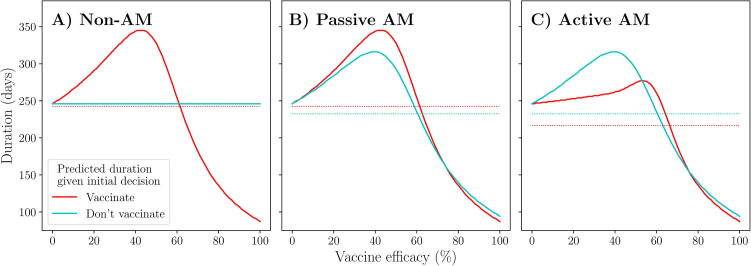
**Scenario 2: Predicted outbreak duration resulting from an initial decision to vaccinate or not.** Predicted outbreak duration from implementing vaccination immediately (red) or not (blue), as viewed under non-AM (A), passive AM (B) and active AM (C) methods. Dotted lines represent the expected duration, calculated over a *Beta*(1.1,1.1) prior distribution around vaccine efficacy (see Appendix A.4). Epidemiological and vaccination parameters are set to those in Table A.2: Scenario 2.

Under passive AM, an initial decision to vaccinate is assumed to always result in continued vaccination after day $t^{*}$, since stopping the campaign results in a higher expected duration over the prior distribution (Fig. 4). Hence, the expected duration from an initial decision to vaccinate is equivalent to the expected duration from an immediate, full campaign (Fig. 5 red line). In contrast to scenario 1, an initial decision not to vaccinate is assumed to result in vaccination from day $t^{*}$, hence leading to a delayed campaign ($V_{t^{*},t_{\mathrm{end}}}$), since this provides a lower expected duration over the prior distribution than not vaccinating throughout the outbreak (Fig. 4). Thus, in making the initial decision under passive AM, we compare the expected duration of an immediate, full campaign and a delayed campaign. In this case, the latter provides the lowest expected duration, as previously explained, hence the optimal policy for passive AM is to not vaccinate initially. Since our initial decision is not to vaccinate, no new information would be available on day $t^{*}$, hence the final decision would be to vaccinate from this day based on the prior distribution, leading to a delayed campaign.

Under active AM, we again recognise that an initial decision to vaccinate can lead to a final decision to continue vaccination, leading to campaign 1: $V_{0,t_{\mathrm{end}}}$, if the success rate of monitored vaccinations is sufficiently high, or stop vaccination, leading to campaign 2: $V_{0,t^{*}}$, if the success rate is low. In this case, at efficacies below approximately 60%, it is more effective to stop vaccination on day $t^{*}$ than continue it. Formally, in this scenario, an initial decision to vaccinate would lead to continued vaccination if there are at least 21 successes from the 35 monitored vaccinations, as this results in a posterior distribution around vaccine efficacy that assigns a lower expected duration to continuing than stopping. If there are less than 21 successful monitored vaccinations, we would stop vaccination on day $t^{*}$. Hence, the expected cost of an initial decision to vaccinate is a combination of campaigns (1) and (2) (Fig. 5 red line). An initial decision not to vaccinate means there are no monitored vaccinations to provide updated information regarding the vaccine efficacy, hence, as under the passive AM approach, the expected duration of such an initial decision is equivalent to that of a delayed campaign (Fig. 5 blue line). For active AM, the optimal policy is to vaccinate initially, since it provides a lower expected duration over the prior distribution, again arising from the recognition that an ineffective campaign can be stopped on day $t^{*}$, reducing the negative effects of such a campaign.

Overall, as in scenario 1, we observe that the three methods of incorporating the information from monitored vaccinations result in different management decisions. Following a non-AM approach, the optimal policy is to vaccinate immediately and continue this until the vaccine pool is depleted. The optimal policy for passive AM is to not vaccinate immediately, but start vaccination on day $t^{*}$ and continue until the vaccine pool is depleted. Finally, the optimal policy for active AM is to start vaccination immediately and continue until the vaccine pool is depleted if at least 21 of the 35 monitored vaccinations are successful, otherwise cease vaccination on day $t^{*}$. By incorporating the possible future results of monitored vaccinations into our initial decision, following an active AM approach leads to an expected duration that is almost 30 days shorter than if we followed a passive AM approach, a decrease of approximately 12%. Again, active AM is therefore the only approach that truly meets our objective to minimise the expected outbreak duration.

### 3.3 Prior information

**Fig. 6:**
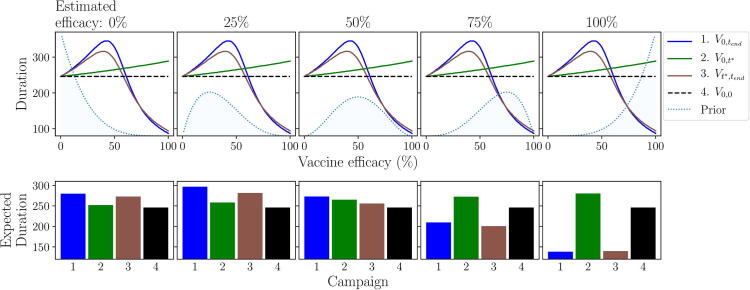
**Example effect of prior information on the expected duration of the outbreak under each campaign.** The expected duration of the outbreak under each campaign is calculated over the prior distribution around vaccine efficacy, defined by a $\mathrm{Beta}(x_{0}+1,y_{0}+1)$ distribution (see Appendix A.4). We set $x_{0}+y_{0}=4$ and vary the estimate of vaccine efficacy (the mode of the distribution $\frac{x_{0}}{x_{0}+y_{0}}$) across columns. Row 1: visual representation of how the prior distribution changes with estimated efficacy. Row 2: expected duration of the outbreak under each campaign for different estimates of vaccine efficacy. Epidemiological and vaccination parameters are set to those in Table A.2: Scenario 2.

**Fig. 7:**
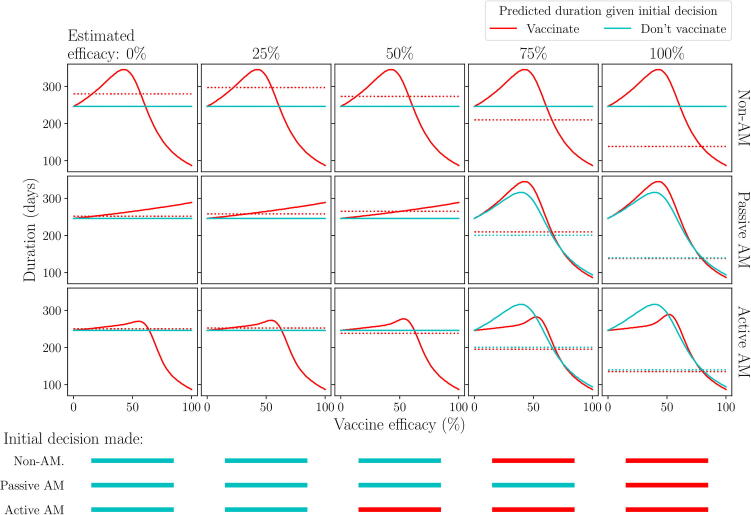
**Example effect of prior information on the expected duration of the outbreak given an initial decision to vaccinate or not.** We set $x_{0}+y_{0}=4$ and vary the estimate of vaccine efficacy (the mode of the distribution $\frac{x_{0}}{x_{0}+y_{0}}$) across columns. Rows 1–3: predicted outbreak duration over vaccine efficacy, given an initial decision to vaccinate (red) or not (blue), for different prior estimates of efficacy, as viewed under a non-AM, passive AM or active AM approach respectively. Bottom row: initial decision made under each approach, for different prior estimates of efficacy: vaccinate (red) or don’t (blue). Epidemiological and vaccination parameters are set to those in Table A.2: Scenario 2.

**Fig. 8:**
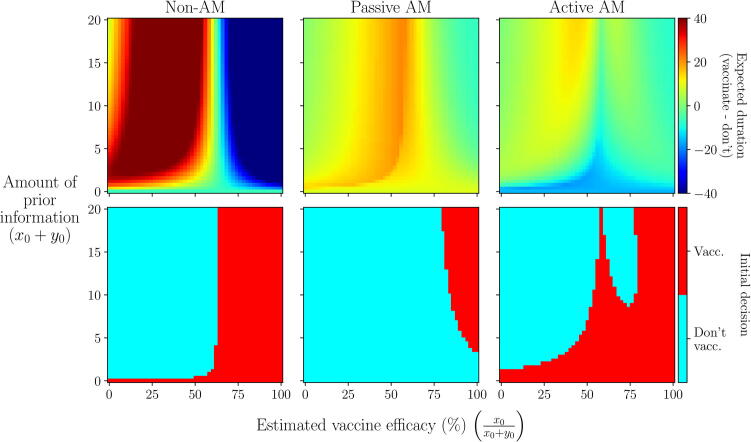
**Scenario 2: Initial decision made under each approach given different prior information.** We define prior information using a Beta($x_{0}+1,y_{0}+1$) distribution (Appendix A.4) and vary the estimated efficacy (the mode of the distribution; $\frac{x_{0}}{x_{0}+y_{0}}$) and the amount of information supporting this estimate ($x_{0}+y_{0}$). Top row: difference in expected duration between vaccinating initially or not, as viewed under each approach. Bottom row: initial decision made under each approach: vaccinate (red) or not (blue). Epidemiological and vaccination parameters are set to those in Table A.2: Scenario 2.

**Fig. 9:**
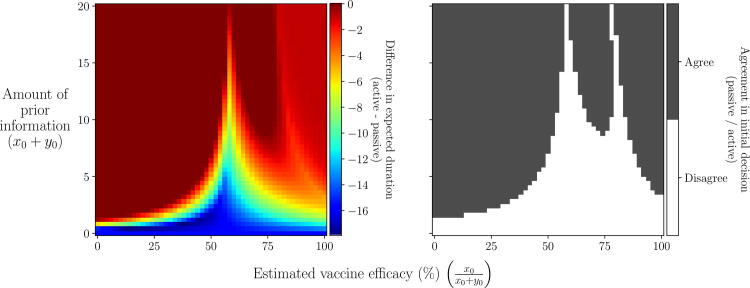
**Scenario 2: Comparison of initial decision made between active and passive AM given different prior information.** We define prior information using a Beta($x_{0}+1,y_{0}+1$) distribution (Appendix A.4) and vary the estimated efficacy (the mode of the distribution; $\frac{x_{0}}{x_{0}+y_{0}}$) and the amount of information supporting this estimate ($x_{0}+y_{0}$). Left panel: difference in expected duration under active AM compared to passive AM. Right panel: agreement in initial decision between passive AM and active AM. Epidemiological and vaccination parameters are set to those in Table A.2: Scenario 2.

Thus far, in both scenarios, we have assumed a very low level of prior information regarding the efficacy of the vaccine, defined by a Beta(1.1,1.1) distribution (see Appendix A.4). If we increase the amount of prior information available, in either scenario, it becomes more likely that the approaches will make the same initial decision, since the information gained from monitored vaccinations has relatively less impact. Which choice is made, to vaccinate initially or not, depends on the estimate of efficacy that is suggested by the prior information (the mode of the distribution $\frac{x_{0}}{x_{0}+y_{0}}$) and the amount of information supporting this estimate ($x_{0}+y_{0}$). In this section, we focus on the effect of changing the prior information under scenario 2 (Fig. 6, Fig. 7, Fig. 8, Fig. 9), however similar conclusions can be drawn from scenario 1, for which the results are provided in Appendix B (Fig. B.16, Fig. B.17, Fig. B.18).

Changing the prior distribution affects the expected duration of all vaccination campaigns, except the ‘no vaccination’ campaign (Fig. 6). This in turn affects the expected outcome of both an initial decision to vaccinate and an initial decision to not vaccinate (Fig. 7).

If the estimate of efficacy provided by the prior information (columns in Fig. 6, Fig. 7, x-axis in Fig. 8) is low, more weight is given to the predicted duration at low efficacies, hence foregoing vaccination entirely becomes the optimal campaign. If there is enough information supporting this estimate, an initial decision not to vaccinate is chosen by all approaches and, since there are no vaccinations to monitor, the choice not to vaccinate will continue throughout the outbreak. The amount of information required for this to occur depends on the approach used (Fig. 8). Under passive AM, even with no prior information (a flat prior distribution over vaccine efficacy) we would choose not to vaccinate. Under a non-AM approach, we require only a very small amount of prior information suggesting efficacy is low to switch from vaccinating to not vaccinating. Finally, under active AM we require slightly more information supporting a low estimate of efficacy ($x_{0}+y_{0}>2$) to make the same switch, since it recognises the possibility that monitored vaccinations may reveal the vaccine efficacy to be higher than estimated.

As the prior estimate of vaccine efficacy increases, more weight is given to the predicted duration of the campaigns at higher efficacies, hence both the immediate, full (1: $V_{0,t_{\mathrm{end}}}$) and delayed (3: $V_{t^{*},t_{\mathrm{end}}}$) campaigns become more effective under the prior distribution (Fig. 6). As a result, the expected duration from an initial decision not to vaccinate switches from being based on no vaccination to delayed vaccination for both passive and active AM (Fig. 7). Since an immediate, full vaccination campaign only results in a shorter outbreak for very high vaccine efficacies (>~80%), compared to a delayed campaign, under passive AM we require a significant amount of prior information supporting an estimate this high to change our initial decision (Fig. 8). Under active AM, however, at low efficacies the shorter predicted duration resulting from vaccinating until day $t^{*}$ and stopping if monitored vaccinations are unsuccessful (campaign 2: $V_{0,t^{*}}$), compared to a delayed campaign that must continue until all vaccines are used, allows the expected duration of an initial decision to vaccinate remain lower than not vaccinating. If vaccine efficacy is very high (>~80%) an immediate, full campaign is optimal, hence estimates in this range also result in an initial decision to vaccinate. Only if there is strong prior information supporting an estimate of efficacy between approximately 55% and 75% will we opt not to vaccinate initially under active AM, since between these values a delayed campaign is optimal (Fig. 4).

The degree of agreement between passive and active AM depends heavily on the prior estimate of efficacy and the strength of information supporting this estimate (right-hand panel; Fig. 9). For any estimate of efficacy, we require at least $x_{0}+y_{0}>2$ for the approaches to agree. This equates to having the amount of information that two monitored vaccinations would provide, prior to the outbreak beginning. For some estimates, such as around 55% and 80%, we require a very large amount of prior information ($x_{0}+y_{0}>20$) for the approaches to agree, since, at these points, the rank of the campaigns cross over causing uncertainty as to which choice is truly optimal. This can result in significantly different expected durations between the two approaches, especially for estimates around 55% where there is still a relatively high possibility that efficacy is low enough to extend the outbreak duration (left-hand panel; Fig. 9). However, for estimates around 80%, whilst the approaches may differ in initial decision, the expected durations under both are similar, since if vaccine efficacy is high there is only a small difference implementing an immediate, full campaign (1: $V_{0,t_{\mathrm{end}}}$) under active AM and a delayed campaign (3: $V_{t^{*},t_{\mathrm{end}}}$) under passive AM. Hence, a different initial decision does not necessarily lead to a significantly different outcome in terms of the management objective.

Finally, we note that our definition of prior information and requirement that $x_{0},y_{0}⩾0$, allows for at most one mode (or none, in the case of a uniform prior). This excludes distributions with two modes, at 0 and 1, that would result if we allowed $-1⩽x_{0},y_{0}⩽0$. Whilst a polarised belief around vaccine efficacy would be uncommon, it could easily be incorporated into this framework. In this scenario, where $\nu_{e}=0$ results in the outcome of all campaigns converging, the mode at $\nu_{e}=1$ would dominate and immediate, full vaccination would be the obvious choice under all management approaches. In scenario 1 (Fig. B.16, Fig. B.17, Fig. B.18), the campaigns diverge at both extreme values of vaccine efficacy, vary almost linearly between and switch rank at close to 50% efficacy. Hence, our decisions would be very similar to those we obtain under a unimodal distribution, following whichever mode carries the most weight.

Overall, we find that as long as there is still significant uncertainty as to which choice of initial action is best, even with prior information, active AM will result in a lower expected cost than passive AM and is hence the only approach that truly minimises the expected duration of the outbreak given the information and resources available.

### 3.4 Monitoring

**Fig. 10:**
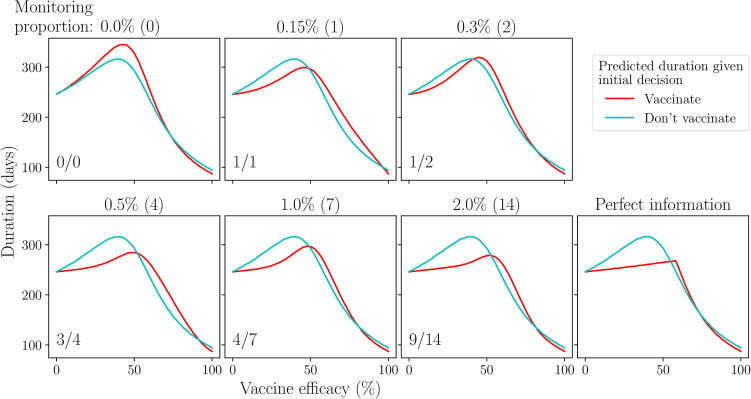
**Scenario 2: effect of monitoring proportion on the predicted outbreak duration given an initial decision to vaccinate or not.** Predicted outbreak duration over vaccine efficacy, given an initial decision to vaccinate (red) or not (blue), for different monitoring proportions (ρ; Table A.2). The number of monitored vaccinations is given in brackets beside the proportion. The required number of successful vaccinations from the total number monitored in order to make a decision to vaccinate is shown in the lower left corner of each panel. The far right panel assumes perfect information is obtained after day $t^{*}$, that is, we will know the true vaccine efficacy exactly when making the final decision. Epidemiological and vaccination parameters are set to those in Table A.2: Scenario 2.

For active AM, the initial control decision depends on the number of vaccinations that are monitored for success. This occurs through the expected outcome given an initial decision to vaccinate ($E[C(a_{v}^{i})]$; Eq. A.16, Appendix A.5), which will depend on how the outcomes of monitored vaccinations affect the posterior distribution around vaccine efficacy (the expected outcome given an initial decision not to vaccinate does not depend on the number of monitored vaccinations since it does not allow monitoring). We explain in detail this effect for scenario 2 (Fig. 10); however analogous statements can easily be made for scenario 1 (Fig. B.19).

First, if no monitoring is planned, active AM views an initial decision to vaccinate in the same way as passive AM, hence assumes that the campaign will always be continued until all vaccines are used since this produces a lower expected duration than stopping the campaign on day $t^{*}$ over the prior distribution around vaccine efficacy. Thus, the expected duration from an initial decision to vaccinate converges to that of an immediate, full campaign at low monitoring proportions (top-left; Fig. 10). In this case, we would make an initial decision not to vaccinate, with the intention of vaccinating from day $t^{*}$ instead, as under passive AM.

As the amount of monitoring increases, the expected duration from an initial decision to vaccinate diverges from that of an immediate, full campaign, becoming a weighted combination of both an immediate, full campaign ($V_{0,t_{\mathrm{end}}}$) and a campaign that is stopped on day $t^{*}$ ($V_{0,t^{*}}$; Fig. 10). This is the result of having monitored vaccinations to inform the final decision: if successes are low, stop the campaign, otherwise continue it. If we have only one monitored trial, we require it to be successful to continue the campaign. Even at low values of vaccine efficacy, there is still a chance that the monitored vaccination will be successful, hence the campaign may be continued when it should not be. The opposite is true at high values of efficacy. As a result, the predicted duration does not coincide exactly to either of the two campaigns that we can choose from, but rather a weighted average of the two. With two monitored vaccinations, we require only one of the two to be successful to continue the campaign. As the number of monitored vaccinations continues to increase, the required number of successes approaches 60% of the total, as this is the value of efficacy at which continuing the campaign becomes more effective than stopping it.

With more trials, the probability of making an incorrect final decision decreases. That is, there is less chance of achieving higher than 60% successes if the true vaccine efficacy is actually below this, and vice versa. As a result, the predicted duration from initial decision to vaccinate more closely approximates a stopped campaign ($V_{0,t^{*}}$) at low efficacies and a full campaign ($V_{0,t_{\mathrm{end}}}$) at high efficacies. Only at efficacy values close to 60% do we still see a significant divergence from both. If we were to assume that monitoring provided perfect information, as is the case when using metrics such as the Expected Value of Perfect Information (EVPI) (Shea et al., 2014, Bradbury et al., 2017), we assume that we always make the correct final decision. This is equivalent to the posterior distribution of vaccine efficacy being a single point at the true value and results in a predicted duration from an initial decision to vaccinate coinciding exactly with either the immediate, full campaign or stopped campaign, with no divergence even when close to the true efficacy (bottom-right; Fig. 10).

**Fig. 11:**
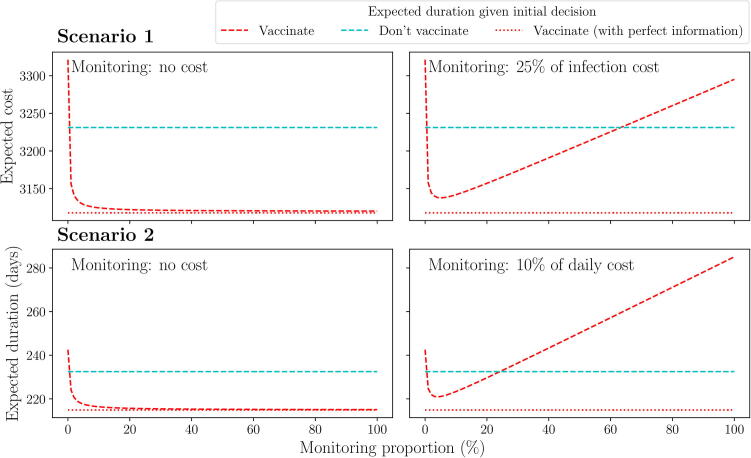
**Effect of monitoring proportion on the expected cost/duration given an initial decision to vaccinate or not.** Top row: expected cost (scenario 1) given an initial decision to vaccinate (red) or not (blue) for a range of monitoring proportions, with and without a cost associated with monitoring (right and left panel respectively). ‘25% of infection cost’ refers to the cost assigned to monitoring a single vaccination, relative to the cost of a single infection. Bottom row: same as top row for scenario 2. The dotted red line represents the expected cost/duration given an initial decision to vaccinate, assuming monitored vaccinations provide perfect information after day $t^{*}$.

Overall, the effect of having more monitoring information reduces the probability of making an incorrect final decision. For both scenarios, this will lower the expected cost or duration from an initial decision to vaccinate towards that provided by perfect information (Fig. 11). We require only one monitored vaccination to make learning about vaccine efficacy worthwhile, allowing an initial decision to vaccinate to become the optimal decision. We also see that, whilst we can always allocate more resources to monitoring to lower the expected outcome towards that provided by perfect information, the effect of doing so decreases and becomes negligible after approximately 70 monitored vaccinations (ρ=10%). If we were to assign a cost to monitoring itself, there would be a point at which adding more monitoring would cost more than it was worth, leading to a single minima which active AM can be used to find (right-hand column; Fig. 11). For scenario 1, a cost per monitored vaccination equivalent to 25% of the cost of an infection results in an optimal monitoring proportion of 5%. Similarly, for scenario 2, a cost per monitored vaccination equivalent to 10% of the daily cost of the outbreak results in an optimal monitoring proportion of 5%. As the cost of monitoring increases, the optimal monitoring proportion will clearly fall and the best attainable outcome (expected cost or duration) will rise (Fig. B.20). If the monitoring cost is high enough, an initial decision to vaccinate may longer be optimal.

### 3.5 Restrictions on control

**Fig. 12:**
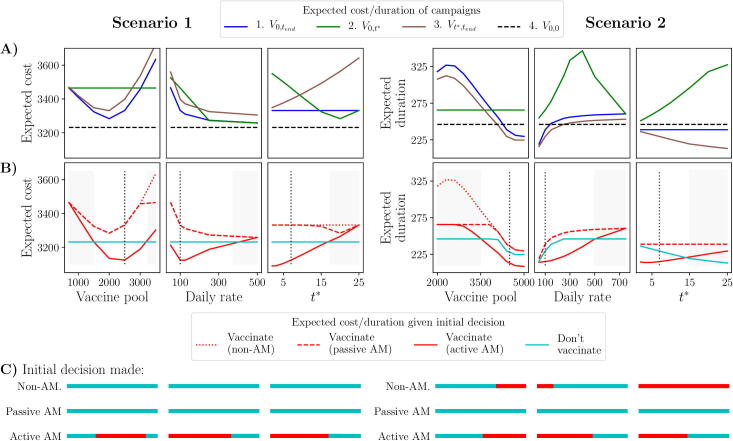
**Sensitivity of results to restrictions on control.** Varying the vaccine pool ($\nu_{\mathrm{pool}}$), daily vaccination rate ($\nu_{r}$) and length of the monitoring period ($t^{*}$), we display the change in expected cost/duration given both the campaigns (row 1) and initial decision under each approach (row 2). The bottom row displays the initial decision made under each approach for different values of these parameters. Results for scenario 1 are displayed on the left and scenario 2 on the right. Parameters a varied one at a time, keeping all others constant at the values provided in Table A.2. Vertical dotted lines identify the default parameter values used throughout. Areas of the parameter space for which passive and active AM agree in their initial decisions are shaded grey.

The vaccination campaigns are defined by a fixed daily vaccination rate ($\nu_{r}$), finite vaccine pool ($\nu_{\mathrm{pool}}$) and a single day on which real-time information can be used to adapt control ($t^{*}$). In both scenarios, these conditions have so far been fixed (Table A.2); however they have a significant effect on the decisions made by our approaches, causing the outcomes to be trivial in some cases and complex in others (Fig. 12).

First, we vary the vaccine pool and keep the other two control restrictions constant (1st and 4th columns; Fig. 12). In both scenarios, if the vaccine pool is too small, we will choose to forego vaccination for the entirety of the outbreak. In scenario 1, this is due to the cost of implementing the vaccination campaign outweighing the number of infections avoided, and in scenario 2, because administering such a small number of vaccines is likely to increase the duration of the outbreak even with a highly effective vaccine (Fig. B.21). In scenario 1, we see that a very large vaccine pool ($\nu_{\mathrm{pool}}>3200$) will also cause us to forego vaccination, since the relative effect of each vaccine, in terms of the reduction in the number of infections each causes, is diminished so much that the campaign is no longer cost effective (Fig. B.21). However, if the vaccine pool is neither too small nor too large to make the initial decision obvious, our approaches will lead to different decisions. Furthermore, we note that, if we were not to fix the vaccine pool, but rather try to optimise its size, only active AM could be relied on to do so. This is clear in the case of scenario 1: active AM can clearly identify that a vaccine pool size of 2500 leads to the lowest expected cost from the outbreak, since it minimises the expected cost of an initial decision to vaccinate (1st column; Fig. 12). However, both passive and non-AM would suggest that 2000 vaccines is the optimal pool size, if they were to vaccinate, since they are biased by the high cost of an ineffective campaign, which is avoided under active AM since we recognise that an ineffective campaign can be stopped before the vaccine pool is depleted.13.

**Fig. 13:**
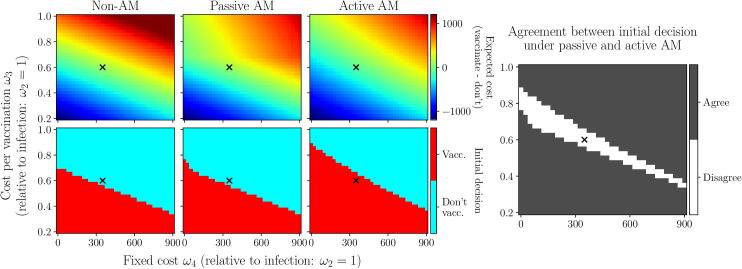
**Scenario 1: initial decisions given different relative costs associated with vaccinations and infections.** We vary the cost per vaccination ($\omega_{3}$) and fixed cost associated with implementing a campaign ($\omega_{4}$), relative to the cost per infection ($\omega_{2}=1$; see Appendix A.3). Left-hand panels: difference in expected cost between vaccinating initially or not, as viewed under each approach (top row), and initial decision made under each approach. Right-hand panel: agreement in initial decision between passive AM and active AM. Black crosses represent the default values used in scenario 1 (Table A.2).

Next, we vary the daily vaccination rate, keeping the vaccine pool size and $t^{*}$ constant (2nd and 5th columns; Fig. 12). For both scenarios, a high daily rate highlights no vaccination as the obvious choice, although for slightly different reasons. In scenario 1, a high daily rate improves the effectiveness of all vaccination campaigns, however the benefit of being able to stop a campaign that is ineffective is removed, hence we can no longer exploit this through active AM (Fig. B.22). In contrast, in scenario 2, a higher daily rate worsens our campaigns, since the negative effects of vaccination (increased duration at low vaccine efficacy) are exaggerated. Alongside this, the benefit of stopping an ineffective campaign under active AM is again reduced, hence the obvious decision becomes to not vaccinate (Fig. B.22). However, if the daily vaccination rate is not too large (scenario 1: <400, scenario 2: <500), the approaches will lead to different initial decisions. Again, we note that if we wanted to optimise the daily rate rather than assume it fixed, active AM is the only approach that can do so. This is highlighted in scenario 1: under active AM we identify a daily rate of approximately 100 per day as the optimal (2nd column; Fig. 12), allowing learning about vaccine efficacy without committing too many vaccines early on. However, under a non-AM or passive AM approach we would opt to vaccinate as quickly as possible.

Finally, we vary the day on which we use the results from monitored vaccinations to adapt control ($t^{*}$), keeping the vaccine pool size and daily rate constant (3rd and 6th columns; Fig. 12). For both scenarios, if this day is too far in the future, an initial decision not to vaccinate becomes the obvious optimal choice. For scenario 1, this will lead to a final decision also not to vaccinate, caused by the fact that the benefit of stopping an ineffective campaign is removed (as with a high daily vaccination rate), since most of the vaccine pool will have already been used. In scenario 2, the benefit of stopping an ineffective campaign is also removed, but the effectiveness of a delayed campaign is also increased due to a longer delay (Fig. B.23). As a result, in this scenario high values of $t^{*}$ lead to the implementation of a delayed campaign under both adaptive approaches, and an immediate, full campaign under a non-AM approach. If we wished to optimise the length of this delay in scenario 2, under a passive AM approach we would choose to make the delay as long as possible, to optimise a delayed campaign, whereas under active AM we could identify a better optimal value for $t^{*}$ of around 5 days (6th column; Fig. 12).

### 3.6 Management objective

It is clear from the contrast between scenarios 1 and 2 that the management objective has a significant impact on the decisions made under any of the three approaches. Furthermore, in scenario 1, the relative costs of infections compared to vaccinations will also have such an influence. If the costs of vaccination (both per vaccine costs, $\omega_{3}$, and a fixed cost associated with implementing a vaccination campaign, $\omega_{4}$; Appendix A.3) are sufficiently high, an initial decision to vaccinate will not be deemed optimal under any approach. Similarly, if these vaccination costs are sufficiently low, vaccination becomes the obvious choice and we will choose to vaccinate under all approaches. However, there is a region in which the choice is not so obvious, where the costs of vaccination may be outweighed by the reduction in infections if the vaccine is effective, but may not if it is ineffective. It is in this region that the initial decision differs between approaches: under active AM we choose to vaccinate and thereby learn about the vaccine efficacy, allowing greater reduction in infections if vaccine efficacy is high, but under the non-AM or passive AM approaches we are unable to foresee the greater worth of doing this and therefore choose not to vaccinate at all.

### 3.7 Epidemiological parameters

The dynamics of the epidemic itself can also render the decision making problem trivial or highly complex. For example, in scenario 1, if $R_{0}$ is less than 1, the epidemic will die out very quickly by itself and hence it is clearly not worth incurring the cost of implementing a vaccination campaign, so under all approaches we would choose not to vaccinate. However, if $R_{0}>1$, we see that only under active AM do we choose to vaccinate (Fig. B.24).

**Fig. 14:**
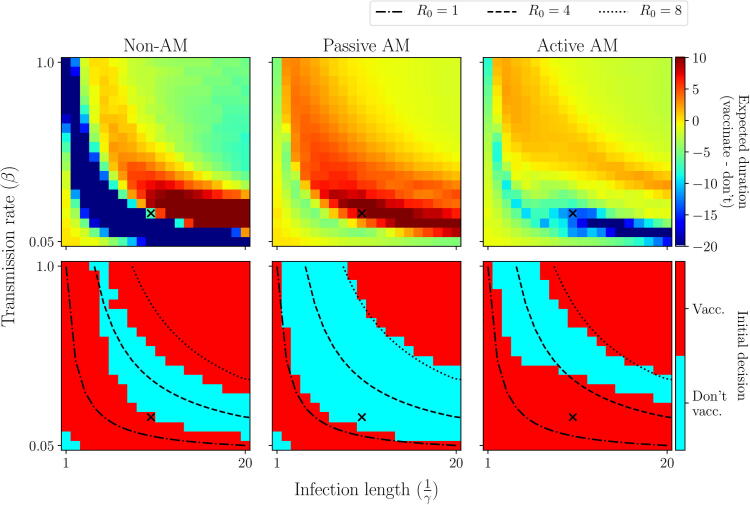
**Scenario 2: Initial decision made under each approach, varying epidemiological parameters.** We vary the epidemiological parameters describing transmission (β) and recovery/removal (γ). Top row: difference in expected duration between vaccinating initially or not, as viewed under each approach. Bottom row: initial decision made under each approach: vaccinate (red) or not (blue). Black crosses represent the default values used in scenario 2 (Table A.2). Lines of constant $R_{0}$ are identified with black lines.

**Fig. 15:**
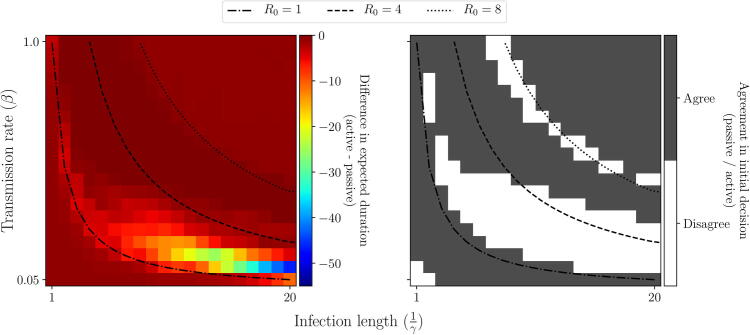
**Scenario 2: Comparison of initial decision made between active and passive AM given different epidemiological parameters.** We vary the epidemiological parameters describing transmission (β) and recovery/removal (γ). Left panel: difference in expected duration under active AM compared to passive AM. Right panel: agreement in initial decision between passive AM and active AM. Black crosses represent the default values used in scenario 2 (Table A.2). Lines of constant $R_{0}$ are identified with black lines.

**Fig. B.16:**
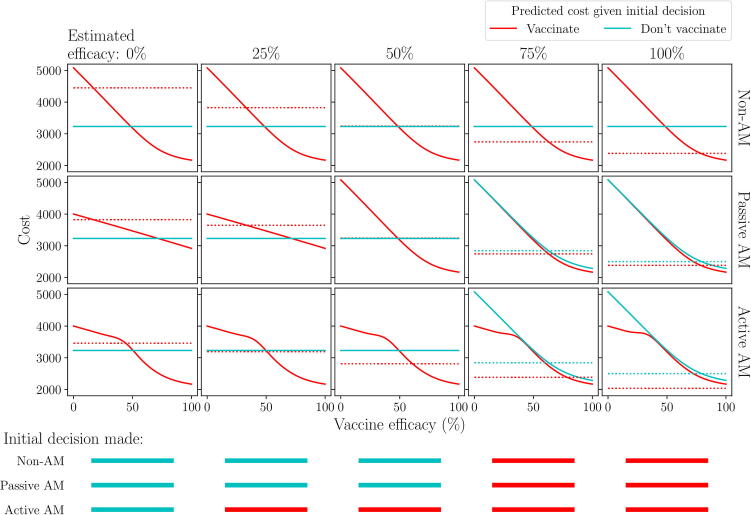
**Example effect of prior information on the expected cost of the outbreak given an initial decision to vaccinate or not.** We set $x_{0}+y_{0}=4$ and vary the estimate of vaccine efficacy (the mode of the distribution $\frac{x_{0}}{x_{0}+y_{0}}$) across columns. Rows 1–3: predicted outbreak cost over vaccine efficacy, given an initial decision to vaccinate (red) or not (blue), for different prior estimates of efficacy, as viewed under a non-AM, passive AM or active AM approach respectively. Bottom row: initial decision made under each approach, for different prior estimates of efficacy: vaccinate (red) or don’t (blue). Epidemiological and vaccination parameters are set to those in Table A.2: Scenario 1.

**Fig. B.17:**
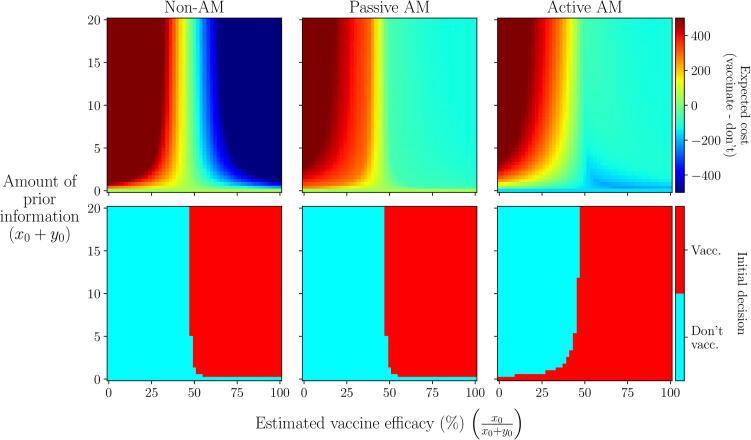
**Scenario 1: Initial decision made under each approach given different prior information.** We define prior information using a Beta($x_{0}+1,y_{0}+1$) distribution and vary the estimated efficacy (the mode of the distribution; $\frac{x_{0}}{x_{0}+y_{0}}$) and the amount of information supporting this estimate ($x_{0}+y_{0}$). Top row: difference in expected cost between vaccinating initially or not, as viewed under each approach. Bottom row: initial decision made under each approach: vaccinate (red) or not (blue). Epidemiological and vaccination parameters are set to those in Table A.2: Scenario 1.

**Fig. B.18:**
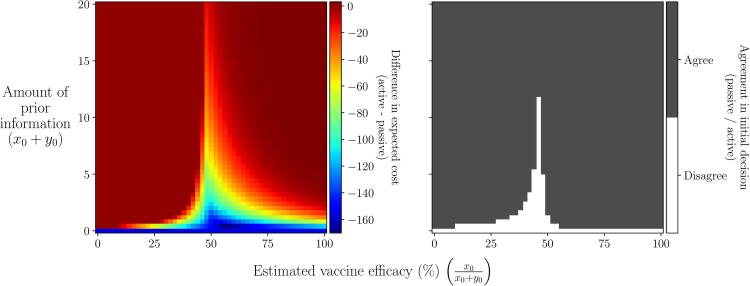
**Scenario 1: Comparison of initial decision made between active and passive AM given different prior information.** We define prior information using a Beta($x_{0}+1,y_{0}+1$) distribution and vary the estimated efficacy (the mode of the distribution; $\frac{x_{0}}{x_{0}+y_{0}}$) and the amount of information supporting this estimate ($x_{0}+y_{0}$). Left panel: difference in expected cost under active AM compared to passive AM. Right panel: agreement in initial decision between passive AM and active AM. Epidemiological and vaccination parameters are set to those in Table A.2: Scenario 1.

**Fig. B.19:**
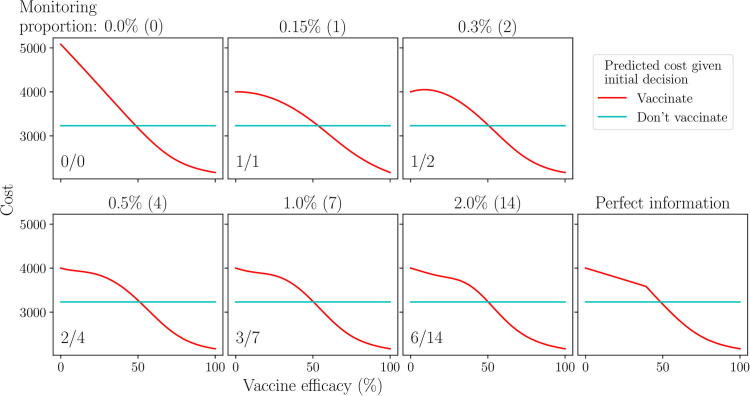
**Scenario 1: effect of monitoring proportion on the predicted outbreak cost given an initial decision to vaccinate or not.** Predicted outbreak cost over vaccine efficacy, given an initial decision to vaccinate (red) or not (blue), for different monitoring proportions (ρ; Table A.2). The number of monitored vaccinations is given in brackets beside the proportion. The required number of successful vaccinations from the total number monitored in order to make a decision to vaccinate is shown in the lower left corner of each panel. The far right panel assumes perfect information is obtained after day $t^{*}$, that is, we will know the true vaccine efficacy exactly when making the final decision. Epidemiological and vaccination parameters are set to those in Table A.2: Scenario 1.

**Fig. B.20:**
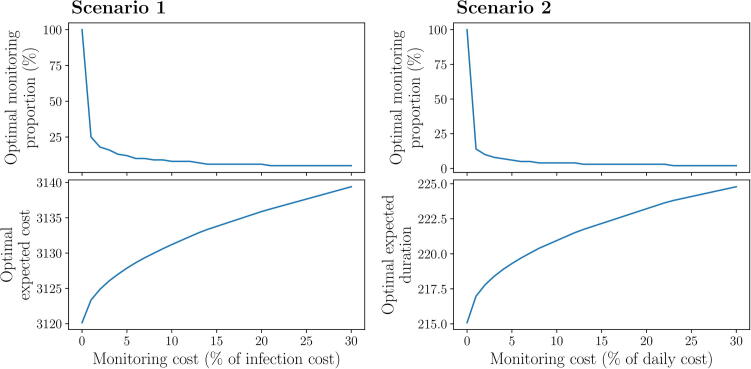
**Effect of cost associated with monitoring on the optimal monitoring proportion and optimal expected cost/duration given an initial decision to vaccinate.** Top row: optimal monitoring proportion given an initial decision to vaccinate for a range of monitoring costs, for scenario 1 (left) and scenario 2 (right). Bottom row: optimal expected outcome (cost or duration) given an initial decision to vaccinate for a range of monitoring costs.

**Fig. B.21:**
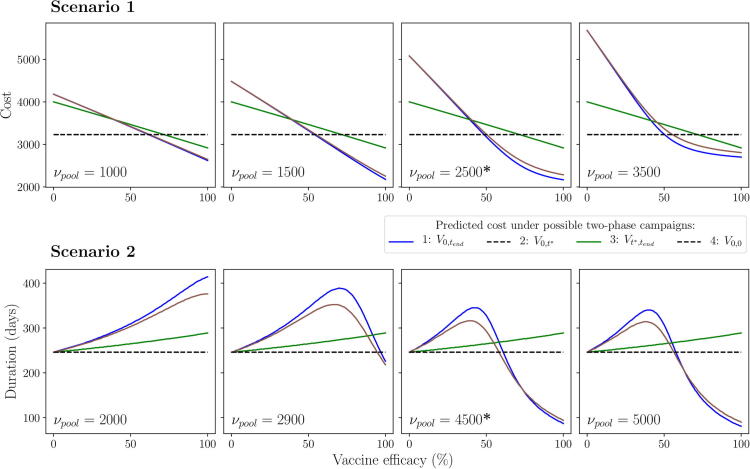
**Effect of vaccine pool size on campaign performance – Scenarios 1 and 2.** All other parameters are fixed to the values given in Table A.2. Asterisks identify the default value for the vaccine pool size used in each scenario.

**Fig. B.22:**
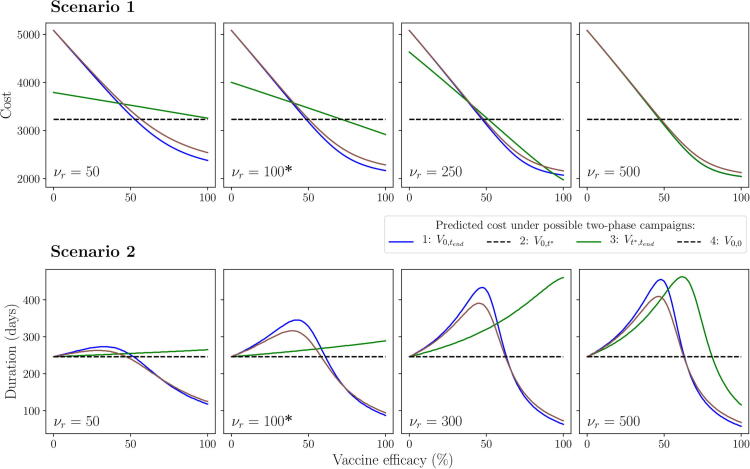
**Effect of the daily vaccine rate on campaign performance - Scenarios 1 and 2.** All other parameters are fixed to the values given in Table A.2. Asterisks identify the default value for the daily rate used in each scenario.

**Fig. B.23:**
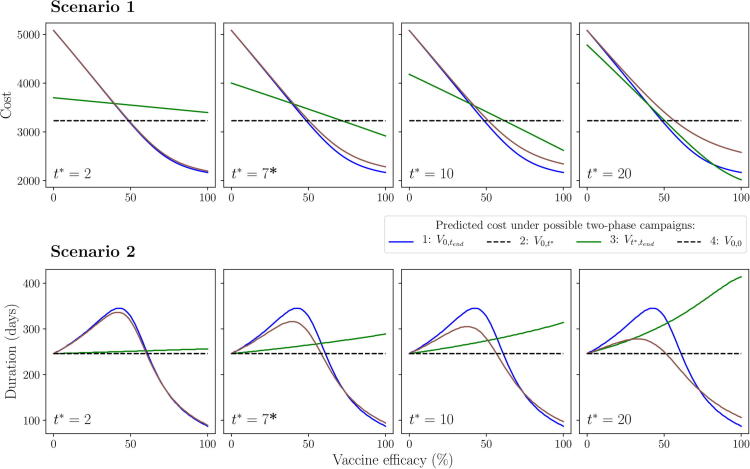
**Effect of the value of**$t^{*}$**on campaign performance – Scenarios 1 and 2.** All other parameters are fixed to the values given in Table A.2. Asterisks identify the default value for $t^{*}$ used in each scenario.

**Fig. B.24:**
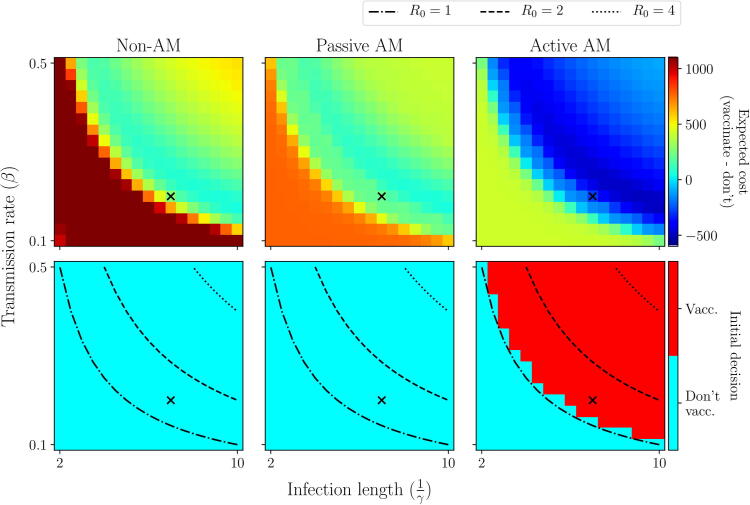
**Scenario 1: Initial decision made under each approach, varying epidemiological parameters.** We vary the epidemiological parameters describing transmission (β) and recovery/removal (γ). Top row: difference in expected cost between vaccinating initially or not, as viewed under each approach. Bottom row: initial decision made under each approach: vaccinate (red) or not (blue). Black crosses represent the default values used in scenario 1 (Table A.2). Lines of constant $R_{0}$ are identified with black lines.

**Fig. B.25:**
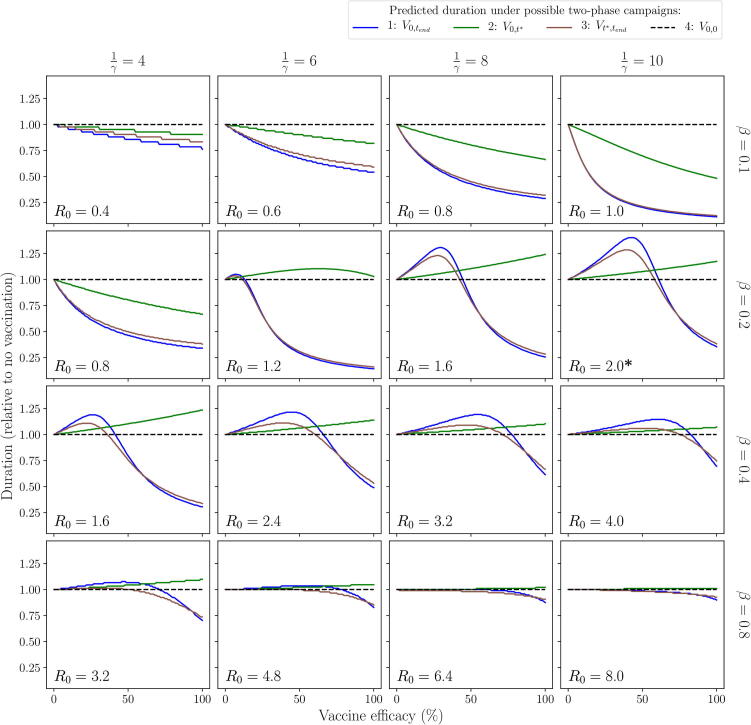
**Effect of epidemiological transmission and recovery rates on campaign performance - Scenario 2.** All other parameters are fixed to the values given in Table A.2. The asterisk identifies the default combination of transmission and recovery rates used in scenario 2.

We see a similar, but more complex, relationship in scenario 2. If $R_{0}$ is very low ($R_{0}<1$) or high ($R_{0}>8$), the negative effects of vaccination (increased duration at low vaccine efficacy) are diminished and hence we would choose to vaccinate under any approach (Fig. 14, Fig. 15). However, between these values, our decision depends on the approach we take. This is most pronounced for $1<R_{0}<4$, with slow recovery rates from infection (long infectious periods). In such circumstances, under passive AM the apparent benefit of a delayed vaccination campaign (under the prior distribution) causes us to make an initial decision not to vaccinate, however this removes our ability to learn. The long infectious period results in an exaggerated negative impact if vaccine efficacy is in fact low. Under active AM however, we recognise this and make an initial decision to vaccinate and learn about efficacy, allowing us to avoid the significant negative impacts of an ineffective vaccine. For epidemics with higher transmission rates and shorter infectious periods, the benefit of a delayed campaign may outweigh the benefit of learning and stopping an ineffective campaign (Fig. B.25).

In reality we will often be dealing with epidemics with an $R_{0}$ in this range ($1<R_{0}<4$), for example Ebola, seasonal influenza, cholera, plague, Zika, to name a few. Only rarely will a disease have an $R_{0}$ value significantly higher than this, such as measles, and if the $R_{0}$ is below 1 then it is unlikely to cause a significant outbreak requiring complex control recommendations.

## 4 Conclusion

In this paper we have developed a model to investigate the effectiveness of adaptive management strategies to control outbreaks of infectious diseases, following different approaches to incorporating real-time information regarding the unknown efficacy of a vaccine. Such approaches may be necessary in the context of infectious disease outbreaks, in which resources are limited, so must be used strategically, and the effectiveness of any vaccination campaign at the start of an outbreak may be uncertain.

We have found that, not only the ability to adapt the control of outbreaks in light of new information, but also the ability to foresee such adaptation, can have a significant effect on the recommendations made and the outcome of the epidemic. Both passive and active AM can improve on a non-AM approach by more appropriately timing the introduction of control or stopping an ineffective campaign when necessary. In a two-phase control set-up such as this, should passive and active AM lead to the same initial decision, both management strategies will result in the same outcome. However, because of the way in which the approaches treat an immediate campaign under uncertainty, they may lead to different initial decisions. In both scenarios we analysed, under active AM, the ability to foresee the option of stopping an ineffective campaign if monitored vaccinations are proving unsuccessful significantly lowered the expected cost (or duration) that would result from an initial decision to vaccinate, when compared with passive AM. This led to an initial decision to vaccinate under active AM, whilst under passive AM we would opt not to vaccinate from the start of the outbreak, removing our ability to learn about vaccine efficacy and ultimately increasing the expected cost (duration) of the outbreak. Therefore, under active AM we are better able to meet the objectives of management. This remained the case across all sensitivity analyses, in which the use of active AM was always at least as good, often better, for meeting management objectives as using the other two approaches.

Although the main result in both scenarios could lead to contrasting recommendations when using passive versus active AM, this is highly dependent on the parameters used. Under certain conditions, the uncertainty around vaccine efficacy does not translate into uncertainty regarding control preference. If taking no action becomes the obvious choice, then all approaches could lead to the same decision not to vaccinate. This is found to occur if the vaccine pool is too small for a campaign to have a significantly positive effect, or similarly if the daily vaccination rate is too high, monitoring period too long, or vaccines too expensive. Conversely, immediate, full vaccination may also become the obvious choice if the cost of vaccines is very low compared to the cost of an infection (for scenario 1), or if the $R_{0}$ of the outbreak is very low or high (for scenario 2). It is plausible that, from a public relations point of view, the cost of appearing not to be taking every possible action to curb an outbreak would be considered high enough that not vaccinating initially would never be an option. However, this highlights another of the benefits of active AM: it provides a complete, evidence based plan of action for all stages of the outbreak, providing a clear outline for control recommendations conditional on different monitoring outcomes and the effect each will have on our ability to satisfy management objectives. Access to this information makes it easier to justify tough, and possibly unintuitive, decisions at early stages of the outbreak, if those decisions are shown to significantly improve the outcome of control in the future. Such scenarios are often not obvious from the outset, hence, whilst an active AM approach may not result in a different recommendation to less complex approaches, this cannot be known *a priori*. Therefore, active AM is useful even if just to confirm and provide evidence supporting the obvious choice of action.

The prior information regarding vaccine efficacy also has a significant effect on the recommendations resulting from each approach, and the difference between the results. As the amount of prior information increases, the relative importance of real-time information is reduced, hence we expect the difference between the results from passive and active AM to be less. Intuitively, if prior information suggests efficacy is low, the approaches are more likely to lead to not vaccinating, whereas if the estimate of efficacy is high, the approaches are more likely to decisions to vaccinate. However, if the prior information still leaves uncertainty as to which campaign is optimal, due to a lack of information or the estimate of efficacy being close to where campaigns switch rank, we are likely to see a difference in results between the approaches.

Care should be taken when using prior information alongside real-time information, since, if given too much weight (i.e. the variance of the prior distribution is too low), it can render the latter redundant. If prior information has been taken from previous outbreaks, it may be inaccurate and hence lead to suboptimal management. For example, in scenario 2, if prior information suggests that vaccine efficacy is between 60–80%, but it is actually significantly lower, relying heavily on this information may lead to opting for a delayed campaign under both active and passive AM, when not vaccinating is truly optimal. This would cause a significant increase in the duration of the outbreak. However, if we reduce the weight we place on prior information, active AM can incorporate the possibility that the true efficacy may still be low and hence results could suggest to vaccinate immediately, reduce uncertainty and stop the campaign if vaccine efficacy is proving to be lower than expected, thereby avoiding much of the negative impact of an ineffective campaign. .

Under active AM, it is also possible to provide more relevant information to decision-makers regarding the amount of monitoring required and the timing and delivery of the vaccines. If there is a cost associated with monitoring, as we would expect in reality, the use of active AM can help to identify the point at which monitoring no longer provides enough information regarding vaccine efficacy to offset the cost of that monitoring. This helps to avoid wasting resources on monitoring that will not affect the control recommendations, possibly allowing more resources to be allocated to control itself. Similarly, under active AM we can optimise the delivery of control through the vaccine pool size, daily vaccination rate and length of the monitoring period. This would not be possible under the other approaches.

In this paper we have focused upon a relatively simple non-spatial model, with non-specific parameters chosen to mirror common non-fatal, human and livestock diseases. We have additionally only focused upon a single uncertainty upon vaccine efficacy to highlight the interaction between control and learning and demonstrate the utility of active AM. In reality, epidemics are much more complex and there are likely to be multiple interacting uncertainties. For novel outbreaks, we may be unaware of the transmission characteristics in the early stages and therefore would not be able to fix the disease parameters as we have in this work. However, it may still be necessary to introduce a control policy rapidly despite the underlying uncertainty. In such circumstances, we are able to treat these parameters as we have vaccine efficacy, defining a prior distribution, possibly using historical data, and using active AM to guide implementation of an optimal multi-phase control policy that explicitly considers resolution of uncertainty as data are accrued during an outbreak. We would expect the potential of active AM to be even greater in such a scenario, when uncertainty is more prominent and therefore the correct course of action based on prior information alone is less clear. It is certainly true that following an active AM approach to management will never result in a worse outcome compared to following a passive or non-AM approach. However, in order to implement and benefit from such an approach in the real world, greater emphasis must be placed on ensuring the components of the AM framework are in place before making management decisions. That is, policy makers must have a clear idea of the objectives they wish to satisfy, the control options available and the data that is going to be collected throughout the outbreak, before making an initial control decision. This information helps to avoid scenarios in which initial control hinders the resolution of uncertainty and our ability to make optimal control decisions in the future. Although, even if all components are clearly and quantitatively defined, the computational complexity of performing active AM can be a barrier to its implementation in real time, leading to the use of sub-optimal passive or non-AM approaches instead. Tackling this issue is a significant focus of current and future work, with possible solutions in areas such as machine learning (Probert et al., 2019).

Whilst analyses of similar systems exist in the literature, for example (Shea et al., 2014, Moore et al., 2017), this paper has extended on such work in two main areas. First, we have applied the adaptive management methodology specifically to an infectious disease epidemiology context and explored in depth how passive and active AM methods can lead to contrasting recommendations at the start of an outbreak. Also, we have not relied upon metrics that assume the complete resolution of uncertainty, such as EVPI, but rather defined a hypothetical, Bayesian method of uncertainty resolution that allows time-dependent, partial resolution of the uncertainty in vaccine efficacy. The methodology we have introduced in this paper allows for the investigation of relatively unexplored areas in the epidemiological literature, for example the balance of resources between uncertainty resolution and control actions, an area that has received significant attention in the conservation and resource management literature (Moore et al., 2017, Probert et al., 2011, Bogich et al., 2008, Baxter and Possingham, 2011, Grantham et al., 2009) but not for epidemiological interventions. The methodology also allows us to clearly examine the effect that control actions can have on our ability to resolve uncertainty. In the context we have used, the resolution of uncertainty is directly linked to the control action available, as in similar applications in the literature (Runge, 2013), since we are not able to monitor vaccinations without administering them. However, this also applies in contexts where there is not a clear, inherent link between control and monitoring. For example, in the case of uncertain epidemiological parameters, control actions may also indirectly affect the gathering of information by hindering the collection of accurate transmission and recovery information through lower disease prevalence or premature removal. This methodology can be used to exemplify how active AM is able to identify and exploit such effects.

In future work we intend to apply this methodology to more realistic, data driven systems in order to further exhibit the utility of active AM as a tool to help inform policy during real-world outbreaks. In this way, we will develop models that can assist in the development of adaptive intervention policies for novel disease outbreaks, thus helping to reduce the impact of such epidemics in the future.

## CRediT authorship contribution statement

**Benjamin D. Atkins:** Conceptualization, Methodology, Formal analysis, Investigation, Writing - original draft, Writing - review & editing, Visualization. **Chris P. Jewell:** Conceptualization, Writing - review & editing, Supervision, Funding acquisition. **Michael C. Runge:** Conceptualization, Writing - review & editing, Funding acquisition. **Matthew J. Ferrari:** Conceptualization, Writing - review & editing, Funding acquisition. **Katriona Shea:** Conceptualization, Writing - review & editing, Funding acquisition. **William J.M. Probert:** Conceptualization, Writing - review & editing, Funding acquisition. **Michael J. Tildesley:** Conceptualization, Methodology, Writing - original draft, Writing - review & editing, Supervision, Funding acquisition.

## Figures and Tables

**Table A.1:** Components of the AM framework.

**Management objectives**
In order to decide upon the ‘best’ control action in the event of an epidemic outbreak, it is necessary to state the objectives that we wish to satisfy. Examples include minimising the duration of the epidemic, loss of life, the economic cost, or, more realistically, a combination of such factors. Policy makers, in cooperation with stakeholders, must explicitly state these goals *a priori*, in a quantifiable manner (Probert et al., 2011).
**Control options**
A list of possible controls that can be implemented throughout the outbreak. These must also be decided upon by policy makers in cooperation with stakeholders. The AM framework forecasts and evaluates the implementation of these controls in different combinations throughout the outbreak to provide a structured, iterative control recommendation.
**Models of system behaviour**
To predict how the disease may spread and how control actions will interact with this spread, quantitative models of system behaviour must be developed. It is essential that competing models capture the key uncertainties within the system, those that affect the recommended course of action (Shea et al., 2014, Li et al., 2017), as well as represent what is known or agreed about the system as accurately as possible. These models are compared and evaluated for credibility during the outbreak. This component of the framework encompasses an impressive body of literature from the epidemiological community.
**Monitoring plan**
As the epidemic proceeds, it is possible to gather information about its behaviour from epidemiologists in the field and local community groups, such as the number of infections or deaths daily, as well as the effect of interventions, for example the efficacy of vaccines. This allows the evaluation of competing control strategies and models of system behaviour as the outbreak proceeds and is essential for resolving system uncertainty and improving control (Probert et al., 2011, Probert et al., 2018). It is necessary to decide on what information to collect before choosing a control action, as this may restrict the resources available and can affect the utility of some actions.
**Management optimisation**
The management objectives, control actions, models of system behaviour and monitoring plan are used to predict, with a level of uncertainty, the effect of interventions under the different models. How the predictions are used depends on the optimisation method (Probert et al., 2011), including passive, trial-and-error type approaches or active approaches to management in which the resolution of uncertainty is built into the optimisation procedure.
**Implementation and adaptation**
The final stage of the AM process is to implement a control action, based on the steps outlined so far, monitor the outbreak and use real-time outbreak information to adapt future management to better accommodate the specified objectives. This may involve reassessing the credibility of competing models, comparing their predictions to what actually happened. Models that are deemed to be less credible are given a lower weight in the optimisation process than those models that are more credible (Probert et al., 2011).

**Table A.2:** **Summary of parameters and notation used.** Scenario specific values apply throughout unless otherwise stated in the sensitivity sections. Values left blank depend on the vaccination campaign and are calculated as required during the optimisation process.

Notation	Description	Scenario 1 value	Scenario 2 value
β	Transmission rate of disease	0.23	0.2
σ	Incubation rate of disease	0.5	0.2
γ	Recovery/removal rate from disease	0.14	0.1
$\nu_{r}$	Daily vaccination rate (number of individuals)	100	100
$\nu_{e}$	Vaccine efficacy	(0,100)%	(0,100)%
$\nu_{\mathrm{pool}}$	Total number of vaccines available	2500	4500
$t^{*}$	Day on which adaptive approaches can adapt control	7	7
$t_{\mathrm{end}}$	Day on which outbreak ends (duration of the outbreak)	–	–
$V_{t_{0},t_{1}}$	Denotes a vaccination campaign that starts on day $t_{0}$ and ends on day $t_{1}$. We require $t_{0}⩽t_{1}⩽t_{\mathrm{end}}$	–	–
$C(V_{t_{0},t_{1}},\nu_{e})$	Cost of an outbreak (H(t)) with a given vaccination campaign ($V_{t_{0},t_{1}}$) and vaccine efficacy.	–	–
$\omega_{1}$	Weight assigned to the length of the outbreak (per day) in calculation of cost	0	1
$\omega_{2}$	Weight assigned to each infection caused by the outbreak in calculation of cost	1	0
$\omega_{3}$	Weight assigned to each vaccination administered in calculation of cost	0.6	0
$\omega_{4}$	Weight associated with a fixed cost of implementing a vaccination campaign in calculation of cost	350	0
$x_{0}$	Number of successful vaccinations that form prior information regarding vaccine efficacy	0.1	0.1
$y_{0}$	Number of unsuccessful vaccinations that form prior information regarding vaccine efficacy	0.1	0.1
ρ	Proportion of administered vaccines monitored for success	5%	5%
$M_{t}$	Number of vaccines monitored for success until time *t*	–	–
$x_{t}$	Number of successful, monitored vaccinations up to time *t*	–	–
$y_{t}$	Number of unsuccessful, monitored vaccinations up to time *t*	–	–

## Appendix A Methods supplement

### A.2 System representation

The differential equations for the system are:

(A.1) $$\begin{array}{c} \frac{\mathrm{dS}(t)}{\mathrm{dt}}=-\beta S(t)I(t)-\nu_{e}\cdot \nu_{r}(t),\\ \frac{\mathrm{dE}(t)}{\mathrm{dt}}=\beta S(t)I(t)-\sigma E(t),\\ \frac{\mathrm{dI}(t)}{\mathrm{dt}}=\sigma E(t)-\gamma I(t),\\ \frac{\mathrm{dR}(t)}{\mathrm{dt}}=\gamma I(t)+\nu_{e}\cdot \nu_{r}(t), \end{array}$$

where $\nu_{r}(t)$ depends on the current vaccination campaign. We denote the vaccination campaign $V_{t_{0},t_{1}}$, representing a campaign that starts on day $t_{0}$ and ends on day $t_{1}$. If $t_{0}⩽t⩽t_{1}$, we say the campaign is currently ongoing and:

(A.2) $$\nu_{r}(t)=\left\{\begin{array}{cc} \nu_{r}, & \mathrm{if}\;S(t)⩾\nu_{r},\\ S(t), & \mathrm{if}\;0⩽S(t)<\nu_{r},\\ \nu_{\mathrm{pool}}-\int_{t_{0}}^{t}\nu_{r}(s)ds, & \mathrm{if}\;\nu_{\mathrm{pool}}-\int_{t_{0}}^{t}\nu_{r}(s)ds<\nu_{r},\\ 0, & \mathrm{if}\;\int_{t_{0}}^{t}\nu_{r}(s)ds>\nu_{\mathrm{pool}}\text{.} \end{array}\right)$$

If $t<t_{0}$ or $t>t_{1}$, the campaign is not currently ongoing and $\nu_{r}(t)=0$. If no vaccination occurs throughout the epidemic, we denote this by $V_{0,0}$. Note that $\nu_{r}(t)$ may also depend on the vaccine efficacy $\nu_{e}$, through the depletion of *S*; we emphasise this with the notation $\nu_{r}(t,\nu_{e})$ where necessary.

Finally, $t_{\mathrm{end}}$ represents the day on which the outbreak ends and hence the duration of the outbreak (relative to the time of detection). This also depends on the vaccination campaign chosen and vaccine efficacy. Given the continuous nature of the differential equations (Eq. A.1), we define this to be the point at which the number of Exposed and Infectious individuals together falls below 1 (E(t)+I(t)<1).

A summary of parameters and notation used can be found in Table A.2.

### A.3 Management objective

The cost of the outbreak, with a given vaccination campaign ($V_{t_{0},t_{1}}$) and vaccine efficacy ($\nu_{e}$), is calculated using a weighted, linear combination of the duration of the outbreak, the number of vaccines administered, a fixed cost associated with implementing a vaccination campaign and the number of infections caused by the epidemic:

(A.3) $$C(V_{t_{0},t_{1}},\nu_{e})=\omega_{1}\;t_{\mathrm{end}}(\nu_{e})+\omega_{2}\left(\int_{0}^{t_{\mathrm{end}}(\nu_{e})}I(s,\nu_{e})ds\right)+\omega_{3}\left(\int_{t_{0}}^{t_{1}}\nu_{r}(s,\nu_{e})ds\right)+\omega_{4}\;\delta_{V},$$

where

(A.4) $$\delta_{V}=\left\{\begin{array}{c} 1,\;\text{if a vaccination campaign is implemented},\\ 0,\;\mathrm{otherwise}\text{.} \end{array}\right)$$

The cost in scenario 1 is defined by using non-zero weights for $\omega_{2},\omega_{3}$ and $\omega_{4}$ and setting $\omega_{1}=0$. For scenario 2, we let $\omega_{2}=\omega_{3}=\omega_{4}=0$ and $\omega_{1}=1$. The weights, $\omega_{i}$, can either be unitless, representing the relative importance of each, or take a unit such as currency. For example, $\omega_{1}$ may be the daily loss of income to the economy from reduced tourism or exports during the epidemic, $\omega_{2}$ the total cost of care of an infected human or loss of profit from having livestock infected, $\omega_{3}$ the average monetary cost of transporting and administering each vaccine and $\omega_{4}$ the cost of developing a new vaccine and marketing the campaign. The default weights used for the basis of each scenario are given in Table A.2.

The expected cost of the outbreak is calculated by integrating over the probability distribution around vaccine efficacy (Eq. A.5). This distribution, $f(\nu_{e})$, is defined in section A.3.

(A.5) $$E[C(V_{t_{0},t_{1}})]=\int_{0}^{1}C(V_{t_{0},t_{1}},\nu_{e})\cdot f(\nu_{e})\;d\nu_{e}\text{.}$$

We approximate the integral in Eq. A.5 by binning vaccine efficacy into 1% intervals.

### A.4 Prior and real-time information

We define the prior information regarding vaccine efficacy using a *Beta*$\left(x_{0}+1,y_{0}+1\right)$ distribution ($x_{0},y_{0}⩾0$). Hence, the probability density function is:

(A.6) $$f(\nu_{e}\text{;}x_{0},y_{0})=\frac{\Gamma (x_{0}+y_{0}+2)}{\Gamma (x_{0}+1)\Gamma (y_{0}+1)}\nu_{e}^{x_{0}}{\left(1-\nu_{e}\right)}^{y_{0}},$$

where Γ(·) is the Gamma function. The mode of the distribution, $\frac{x_{0}}{x_{0}+y_{0}}$, corresponds to the estimate of vaccine efficacy. The sum $x_{0}+y_{0}$ relates to the amount of information that is supporting the estimate. For example, if the prior information is from a historical vaccine trial, $x_{0}$ could be the number of successful vaccinations and $y_{0}$ the number of unsuccessful vaccinations from the trial. However, to allow non-integer values of $x_{0}$ and $y_{0}$ in our analysis, we define the sum $x_{0}+y_{0}$ to be the relative strength of the prior information compared to a single monitored vaccination (introduced below). If there is no prior information ($x_{0}=y_{0}=0$), the distribution is uniform between 0 and 1 and hence the mode is undefined. For the majority of this analysis, except when explicitly investigating the effect of prior information on our management decisions, we set $x_{0}=y_{0}=0.1$ (Table A.2) when defining our prior distribution around vaccine efficacy. This results in a distribution centred around 50% efficacy with a large variance, representing a situation where we do not have a strong idea of what the efficacy is, but are aware that it is less likely to be completely effective (100% efficacy) or completely ineffective (0% efficacy).

Real-time information is collected throughout the outbreak by monitoring a proportion (ρ) of administered vaccinations for success. We assume that the success or failure of a vaccine can be tested immediately after it is administered and this test will always give the true result. We denote $M_{t}$ as the total number of vaccinations monitored up to time *t*, with $x_{t}$ and $y_{t}$ the number of successful and unsuccessful vaccinations respectively (hence $M_{t}=x_{t}+y_{t}$). This real-time information is combined with the prior information to give a posterior distribution around the vaccine efficacy, defined by a *Beta*$\left(x_{0}+x_{t}+1,y_{0}+y_{t}+1\right)$ distribution with probability density function:

(A.7) $$f(\nu_{e}\text{;}x_{0},y_{0},x_{t},y_{t})=\frac{\Gamma (x_{0}+x_{t}+y_{0}+y_{t}+2)}{\Gamma (x_{0}+x_{t}+1)\Gamma (y_{0}+y_{t}+1)}\nu_{e}^{x_{0}+x_{t}}{\left(1-\nu_{e}\right)}^{y_{0}+y_{t}}\text{.}$$

### A.5 Decision making approaches

We allow a maximum of two decision points (one for the non-AM approach): an initial decision is made when the outbreak is detected (t=0) and a final decision is made on a predetermined day during the outbreak ($t=t^{*}$). We denote the choice to vaccinate or not vaccinate $a_{v}$ and $a_{0}$ respectively, using the superscripts *i* and *f* to represent the initial and final decisions respectively.

#### A.5.1 Non-AM

**Initial decision (**t=0**)** Since this is the only decision point under this approach, a choice to vaccinate now ($a_{v}^{i}$) implies vaccination until vaccine pool depletion and a choice not to vaccinate now ($a_{0}^{i}$) implies forgoing vaccination until the epidemic is over. We compare the two choices by the expected cost of the outbreak resulting from each (Eqs. (A.8), (A.9)), choosing that which produces the lowest expected cost over the prior distribution around vaccine efficacy ($f(\nu_{e}\text{;}x_{0},y_{0})$).

(A.8) $$E[C(a_{v}^{i})]=E[C(V_{0,t_{\mathrm{end}}})]=\int_{0}^{1}C(V_{0,t_{\mathrm{end}}},\nu_{e})\cdot f(\nu_{e}\text{;}x_{0},y_{0})\;d\nu_{e},$$

(A.9) $$E[C(a_{0}^{i})]=E[C(V_{0,0})]=\int_{0}^{1}C(V_{0,0},\nu_{e})\cdot f(\nu_{e}\text{;}x_{0},y_{0})\;d\nu_{e}\text{.}$$

#### A.5.2 Passive AM

**Initial decision (**t=0**)** Since there is a single future decision point at $t=t^{*}$, a choice to vaccinate initially (t=0) implies vaccination until at least $t^{*}$, followed by either vaccination (leading to the campaign $V_{0,t_{\mathrm{end}}}$) or no vaccination ($V_{0,t^{*}}$). Note that we assume the ratio between the number of vaccines available and the daily vaccination rate is such that the vaccine pool will not be depleted before day $t^{*}$. Similarly, a choice not to vaccinate initially can result in forgoing vaccination completely ($V_{0,0}$) or a delayed campaign starting on day $t^{*}$ ($V_{t^{*},t_{\mathrm{end}}}$). When calculating the expected cost of each initial action (Eqs. (A.10), (A.11)), we assume that future decisions will be made optimally, given the information we have. Under passive AM, we do not plan for monitoring, hence we do not incorporate the anticipation of future monitored vaccinations into our initial decision. Instead, we assume that future decisions will be made based on the current level of information, in this case the prior information. Hence, the expected cost of an initial action under passive AM is the minimum of the expected cost of the two campaigns that can result from it, calculated over the prior distribution around vaccine efficacy (Eqs. (A.10), (A.11)).

(A.10) $$\begin{array}{cc} & E[C(a_{v}^{i})]=\underset{t_{1}=\{t^{*},t_{\mathrm{end}}\}}{\min}\left(E[C(V_{0,t_{1}})]\right),\\ & =\underset{t_{1}=\{t^{*},t_{\mathrm{end}}\}}{\min}\left(\int_{0}^{1}C(V_{0,t_{1}},\nu_{e})\cdot f(\nu_{e}\text{;}x_{0},y_{0})\;d\nu_{e}\right), \end{array}$$

(A.11) $$\begin{array}{cc} & E[C(a_{0}^{i})]=\underset{\left(t_{0},t_{1})=\{(0,0),(t^{*},t_{\mathrm{end}})\right\}}{\min}\left(E[C(V_{t_{0},t_{1}})]\right),\\ & =\underset{\left(t_{0},t_{1})=\{(0,0),(t^{*},t_{\mathrm{end}})\right\}}{\min}\left(\int_{0}^{1}C(V_{t_{0},t_{1}},\nu_{e})\cdot f(\nu_{e}\text{;}x_{0},y_{0})\;d\nu_{e}\right)\text{.} \end{array}$$

**Final decision (**$t=t^{*}$**)** If the initial decision was to vaccinate ($a_{v}^{i}$), we make a final decision based on both the prior information and monitored vaccinations. Since there are no more future decision points, this is a simple expectation over two choices: continue vaccination, leading to campaign $V_{0,t_{\mathrm{end}}}$ ($E[C(a_{v}^{i},a_{v}^{f})]$; Eq. A.12), or stop vaccinating, leading to campaign $V_{0,t^{*}}$ ($E[C(a_{v}^{i},a_{0}^{f})]$; Eq. A.13). We choose the option that produces the lowest expected cost over the posterior distribution around vaccine efficacy ($f(\nu_{e}\text{;}x_{0},y_{0},x_{t^{*}},y_{t^{*}})$).

(A.12) $$E[C(a_{v}^{i},a_{v}^{f})]=E[C(V_{0,t_{\mathrm{end}}})]=\int_{0}^{1}C(V_{0,t_{\mathrm{end}}},\nu_{e})\cdot f(\nu_{e}\text{;}x_{0},y_{0},x_{t^{*}},y_{t^{*}})\;d\nu_{e},$$

(A.13) $$E[C(a_{v}^{i},a_{0}^{f})]=E[C(V_{0,t^{*}})]=\int_{0}^{1}C(V_{0,t^{*}},\nu_{e})\cdot f(\nu_{e}\text{;}x_{0},y_{0},x_{t^{*}},y_{t^{*}})\;d\nu_{e}\text{.}$$

If the initial decision was to not vaccinate ($a_{0}^{i}$), no new information has been gained for the final decision, hence we use only the prior information to make our final decision. Again, this is a simple expectation of two choices ($E[C(a_{0}^{i},a_{v}^{f})]$ and $E[C(a_{0}^{i},a_{0}^{f})]$; Eqs. (A.14), (A.15)) and we choose the option that produces the lowest expected cost over the prior distribution around vaccine efficacy ($f(\nu_{e}\text{;}x_{0},y_{0})$). Note that the comparison of these two options has already been performed during the initial decision when calculating the expected cost of an initial decision to not vaccinate (Eq. A.11) and the result remains unchanged.

(A.14) $$E[C(a_{0}^{i},a_{v}^{f})]=E[C(V_{t^{*},t_{\mathrm{end}}})]=\int_{0}^{1}C(V_{t^{*},t_{\mathrm{end}}},\nu_{e})\cdot f(\nu_{e}\text{;}x_{0},y_{0})\;d\nu_{e},$$

(A.15) $$E[C(a_{0}^{i},a_{0}^{f})]=E[C(V_{0,0})]=\int_{0}^{1}C(V_{0,0},\nu_{e})\cdot f(\nu_{e}\text{;}x_{0},y_{0})\;d\nu_{e}\text{.}$$

#### A.5.3 Active AM

**Initial decision (**t=0**)** As under passive AM, a choice to vaccinate initially (from t=0) implies vaccination until at least $t^{*}$, followed by either continued vaccination ($V_{0,t_{\mathrm{end}}}$) or no vaccination ($V_{0,t^{*}}$), and a choice not to vaccinate initially can result in forgoing vaccination completely ($V_{0,0}$) or a delayed campaign starting on day $t^{*}$ ($V_{t^{*},t_{\mathrm{end}}}$). When calculating the expected cost of each initial action ($E[C(a_{v}^{i})]$ and $E[C(a_{0}^{i})]$; Eqs. (A.16), (A.11)), we assume that future decisions will be made optimally, given the information we have. In contrast to passive AM, under active AM we explicitly incorporate the anticipation of results of future monitored vaccinations into our initial decision. Hence, we assume that future decisions will be made based on not only the prior information, but also the results of monitored vaccinations. Since we do not know what the results of monitored vaccinations will be, we take an expectation over all possible results, weighted by the likelihood of observing these results given the prior information we have. For each set of results, we assume that the future decision is made optimally based on the information those results provide. Therefore, the expected cost of an initial action under active AM is the weighted sum of the minimum expected cost of the two campaigns that can result from it, calculated over the posterior distribution around vaccine efficacy for all possible outcomes from monitored vaccinations (Eq. A.16).

(A.16) $$\begin{array}{cc} E[C(a_{v}^{i})]= & \overset{M_{t^{*}}}{\underset{x_{t^{*}=0}}{\sum }}\left(\underset{t_{1}=\{t^{*},t_{\mathrm{end}}\}}{\min}\left(E[C(V_{0,t_{1}})]\right)\right)\cdot f\left(x_{t^{*}}\text{;}M_{t^{*}},x_{0},y_{0}\right),\\ = & \overset{M_{t^{*}}}{\underset{x_{t^{*}=0}}{\sum }}\left(\underset{t_{1}=\{t^{*},t_{\mathrm{end}}\}}{\min}\left(\int_{0}^{1}C(V_{0,t_{1}},\nu_{e})\cdot f(\nu_{e}\text{;}x_{0},y_{0},x_{t^{*}},y_{t^{*}})\;d\nu_{e}\right)\right)\\ & \;\;\cdot f\left(x_{t^{*}}\text{;}M_{t^{*}},x_{0},y_{0}\right), \end{array}$$

where $y_{t^{*}}=M_{t^{*}}-x_{t^{*}}$ and $f\left(x_{t^{*}}\text{;}M_{t^{*}},x_{0},y_{0}\right)$ is the probability density function of a Beta-Binomial distribution with parameters $M_{t^{*}},x_{0}$ and $y_{0}$. Note that with $M_{t^{*}}=0$, this reduces to Eq. A.10, hence, in the absence of any monitored vaccinations, passive and active AM are identical.

Since we only gain updated information if vaccinations have been administered, the expected cost of not vaccinating initially ($E[C(a_{0}^{i})]$) is equivalent under active AM as under passive AM (Eq. A.11).

**Final decision (**$t=t^{*}$**)** Since there are no future decision points, and hence no more opportunities to gather information and adapt control, the final decision under active AM follows the exact same methodology as under passive AM (Eqs. (A.12), (A.13), (A.14), (A.15)), conditional on the initial decision made. Note that, under active AM, we have already performed all the necessary calculations to make this decision. If the initial decision was to vaccinate, we will have monitored $M_{t^{*}}$ vaccinations by day $t^{*}$ and observed a given number of successes and failures, $x_{t^{*}}$ and $y_{t^{*}}$, leading to a posterior distribution around vaccine efficacy. The expected costs of continuing or ceasing vaccination based on this posterior distribution ($E[C(V_{0,t_{\mathrm{end}}})]$ and $E[C(V_{0,t^{*}})]$ respectively) have already been calculated and compared within the calculation of expected cost of an initial decision to vaccinate (Eq. A.16). Thus, rather than recalculating these expected costs, we are able to make the final decision immediately given the number of successful vaccinations on day $t^{*}$. Under passive AM, this would not be possible.

If the initial decision was to not vaccinate, no new information has been gained and hence, as under passive AM, the campaign that produced the lowest expected cost in the calculation of $E[C(a_{0}^{i},a_{0}^{f})]$ (Eq. A.11) would be chosen, without need for recalculation and comparison of these expected costs.

## Appendix B Results supplement

Fig. B.16, Fig. B.17, Fig. B.18, Fig. B.19, Fig. B.20, Fig. B.21, Fig. B.22, Fig. B.23, Fig. B.24, Fig. B.25.
